# Supplementary material for: Effects of Nanopore Confinement on the Conformational, Dynamical, and Self-Assembly Properties of an FG-Repeat Peptide
Source: bioRxiv. 2025 Sep 16:2025.09.11.675621. Preprint. [Version 1] doi: 10.1101/2025.09.11.675621 (PMC12466779; doi:10.1101/2025.09.11.675621)
Supplement: Supplementary file 1 [file media-1.pdf]

Supporting information for:

Effects of Nanopore Confinement on the Conformational, Dynamical, and Self-Assembly Properties of an FG-Repeat Peptide

Wancheng Zhao, Wai-Ming Yau, and Robert Tycko
Laboratory of Chemical Physics
National Institute of Diabetes and Digestive and Kidney Diseases
National Institutes of Health
Bethesda, MD 20892-0520

Figures S1-S20, pages 2-21

Tables S1-S5, pages 22-26

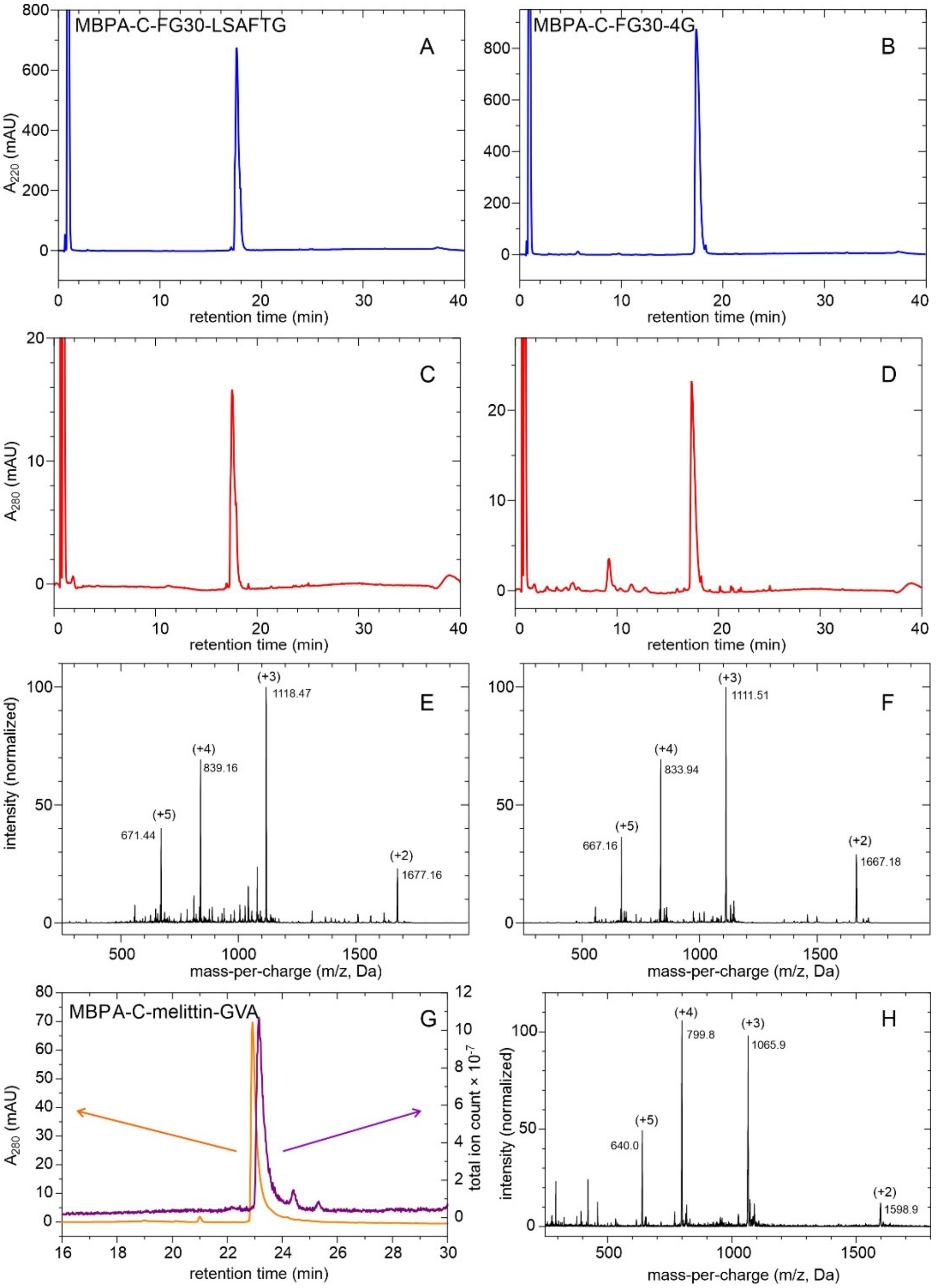

Figure S1: LC-MS data for MBPA-C-FG30-LSAFTG (A,C,E), MBPA-C-FG30-4G (B,D,F), and MBPA-C-melittin-GVA (G,H). Panels A and B show ultraviolet absorption chromatograms at 220 nm. Panels C and D show chromatograms at 280 nm. Panel G shows 280 nm absorption and ion count chromatograms. Panels E, F, and H show electrospray ionization mass spectra for the absorption chromatogram peaks at 17.6 min (E,F) or 30.0 min (H). Mass peaks for ions with charges from +2 to +5 indicate molecular masses of 3352.4 Da for MBPA-C-FG30-LSAFTG, 3331.6 Da for MBPA-C-FG30-4G, and 3195.2 Da for MBPA-C-melittin-GVA. Theoretical values are 3351.5 Da, 3330.5 Da, and 3193.8 Da, respectively.

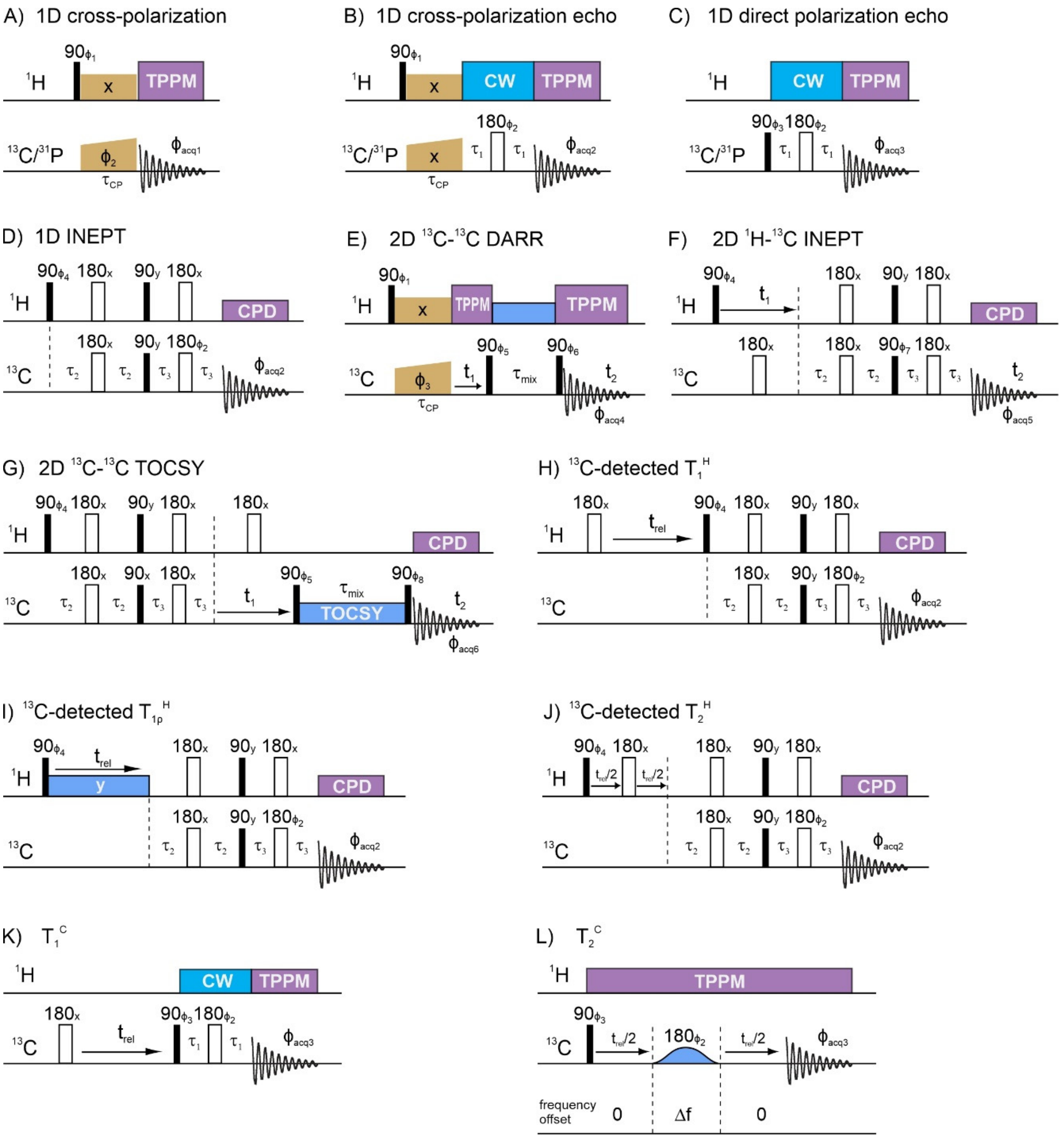

Figure S2: Pulse sequences for all NMR measurements, with TPPM representing two-pulse phase modulated decoupling, CW representing continuous-wave decoupling, and CPD representing composite pulse decoupling using a sequence of 125 phase-shifted π pulses. The same sequence of phase-shifted π pulses was used for ^{13}C-^{13}C mixing in 2D total correlation spectroscopy (TOCSY). Delays in ^{1}H-^{13}C INEPT polarization transfers were $\tau_2 = 1.5$ ms and $\tau_3 = 0.9$ ms. Spin echo delays were $\tau_1 = 78.5$ μs for ^{13}C spectra and $\tau_1 = 76.3$ μs for ^{31}P spectra. Mixing periods were $\tau_{mix} = 30$ ms in 2D ^{13}C-^{13}C DARR measurements and $\tau_{mix} = 12.5$ ms in 2D ^{13}C-^{13}C TOCSY measurements. Frequency offsets Δf were adjusted to select signals of interest in T_2^C measurements, with a selective π pulse length of 2.35 ms (approximate ±10 ppm bandwidth). Non-selective π and π/2 pulse lengths were 8.6 μs and 4.3 μs for ^{1}H, 5.0 μs and 10.0 μs for ^{13}C, and 13.0 μs and 6.5 μs for ^{31}P. Cross-polarization contact times τ_{CP} were optimized for each sample

and were typically 1.2-1.7 ms for ^1H-^{13}C and 0.7-1.1 ms for ^1H-^{31}P. Phase cycling was implemented as follows: ϕ_1 = {y,-y}; ϕ_2 = {x,x,y,y,-x,-x,-y,-y}; ϕ_3 = {x,y}; ϕ_4 = {x,-x}; ϕ_5 = {y,y,-y,-y}; ϕ_6 = {x,x,x,x,y,y,y,y}; ϕ_7 = {x,x,-x,-x,y,y,-y,-y}; ϕ_8 = {x,x,x,x,y,y,y,y,-x,-x,-x,-x,-y,-y,-y,-y}; ϕ_{acq1} = {x,-x,y,-y,-x,x,-y,y}; ϕ_{acq2} = {x,-x,-x,x,x,-x,-x,x}; ϕ_{acq3} = {y,x,-y,-x,y,x,-y,-x}; ϕ_{acq4} = {x,-x,-x,x,y,-y,-y,y}; ϕ_{acq5} = {x,-x,-x,x,-y,y,y,-y}; ϕ_{acq6} = {x,-x,-x,x,y,-y,-y,y,-x,x,x,-x,-y,y,y,-y}.

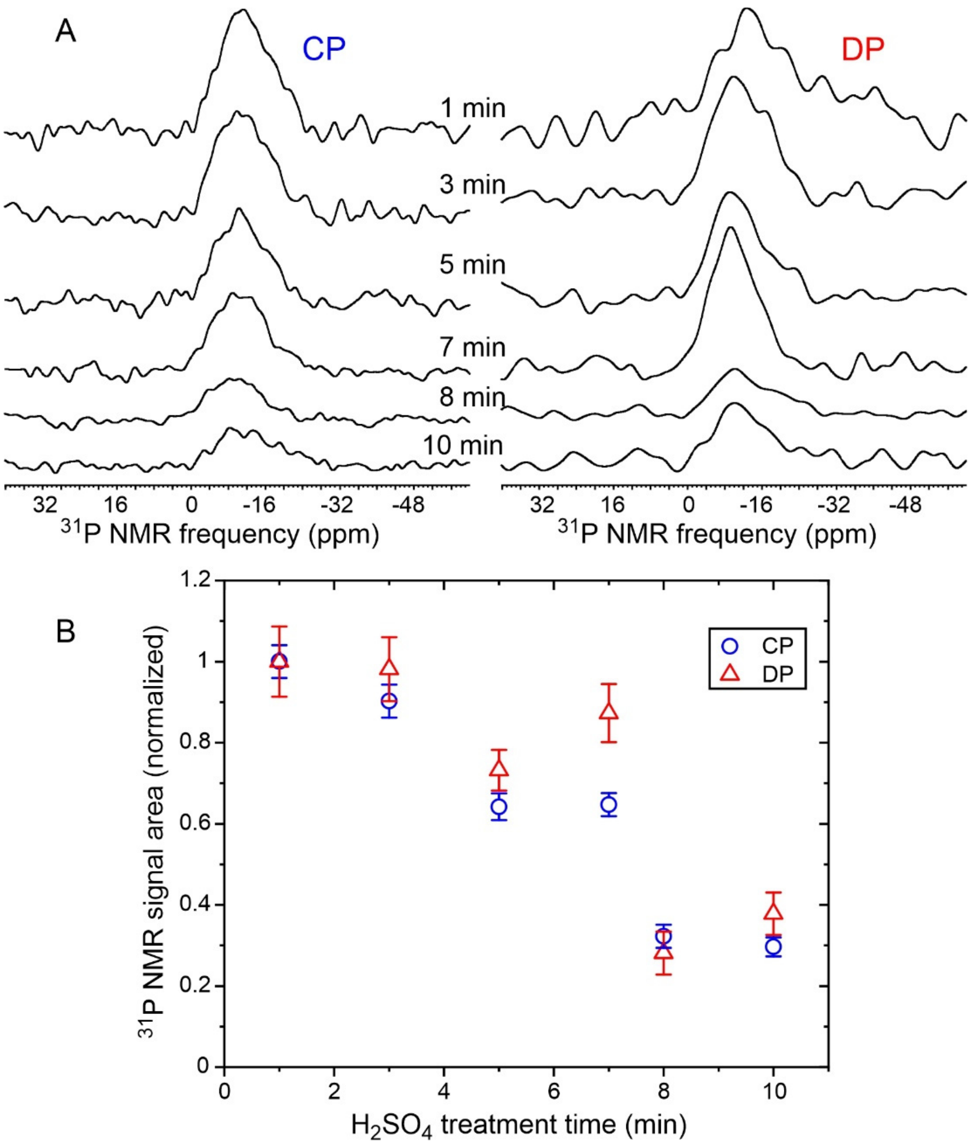

Figure S3: (A) ^{31}P NMR spectra of AAO20 after the indicated treatment times with 0.1 M H_2SO_4, showing the time-dependence of signal from phosphate groups on AAO20 surfaces. Spectra were recorded with ^1H-^{31}P cross polarization (left) or with direct pulsing (right). Vertical scales are adjusted to account for differences in sample masses and signal averaging times. (B) ^{31}P peak areas from spectra in panel A, normalized to 1.0 at 1 min treatment time.

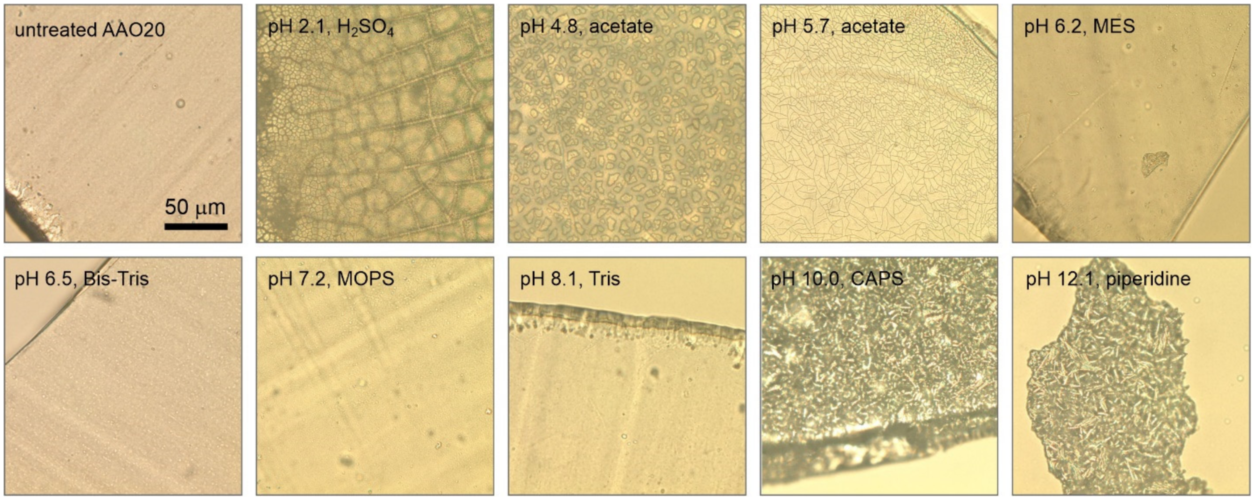

Figure S4: Optical microscope images of AAO20 wafers after incubation for 94 h at 40° C in the indicated buffers (0.2 M buffer concentration, one wafer in 1.5 ml buffer volume, continuous rotation during incubation). Images after incubation at pH values in the 6.2-8.1 range are indistinguishable from images of an untreated wafer. In contrast, incubation at pH values outside this range resulted in obvious damage to the AAO20 material. Incubation at pH 0.4 and pH 14.7 caused complete dissolution of AAO20.

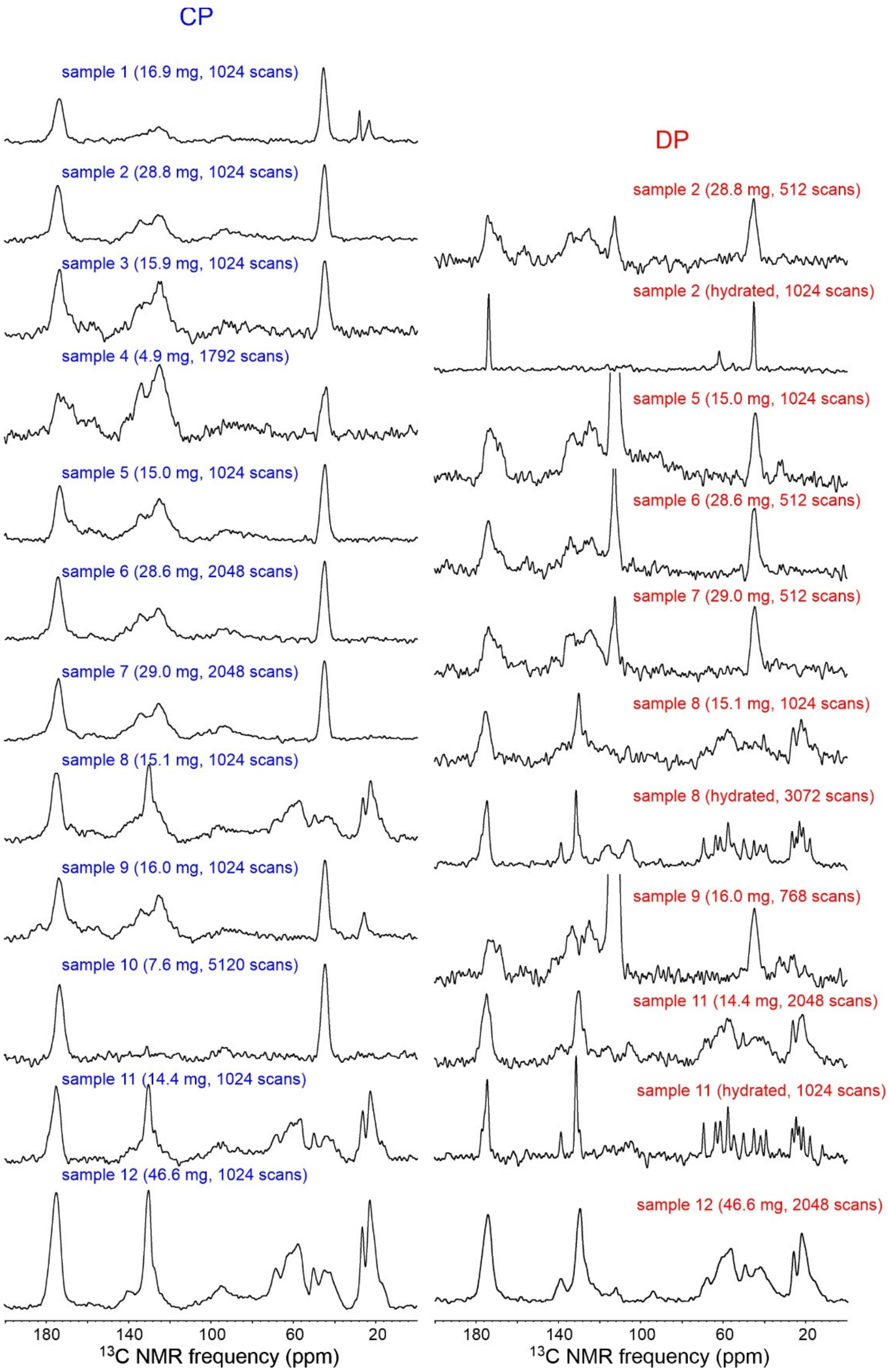

Figure S5: 1D ^{13}C NMR spectra of MBPA-C-FG30/AAO20 samples listed in Table 2 of the main text. Spectra were recorded at 14.1 T with 12.00 kHz MAS and were obtained with ^{1}H-^{13}C cross-polarization (left) or direct pulsing (right). Loading values in Table 2 were determined by comparing aliphatic signal areas in these spectra with aliphatic signal areas in Fig. S7A.

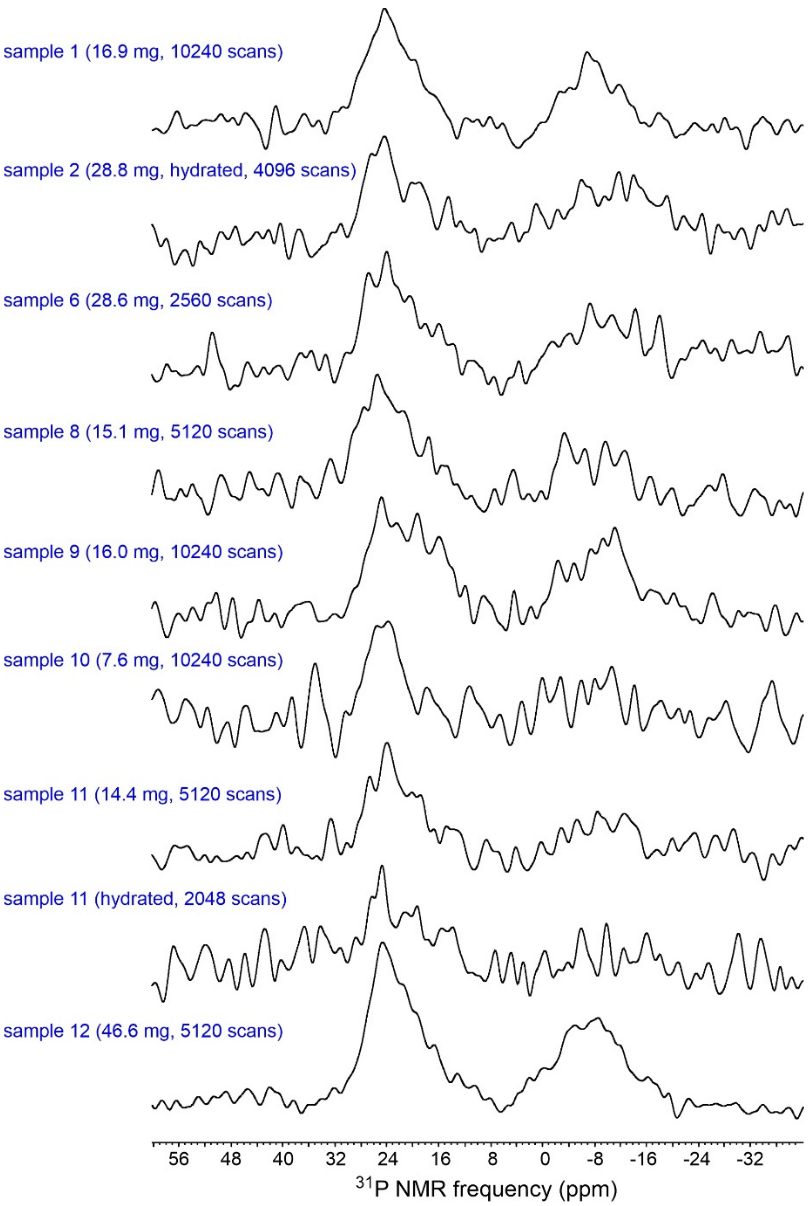

Figure S6: 1D ^{31}P NMR spectra of MBPA-C-FG30/AAO20 samples listed in Table 2 of the main text, obtained with ^{1}H-^{31}P cross-polarization. Loading values in Table 2 were determined by comparing signal areas in the 6-32 ppm range with the signal area in the cross-polarized spectrum in Fig. S7B. Signals upfield of 6 ppm arise from residual phosphate groups on AAO20 surfaces that were not fully removed by the treatment with 0.1 M H_2SO_4 shown in Fig. S3.

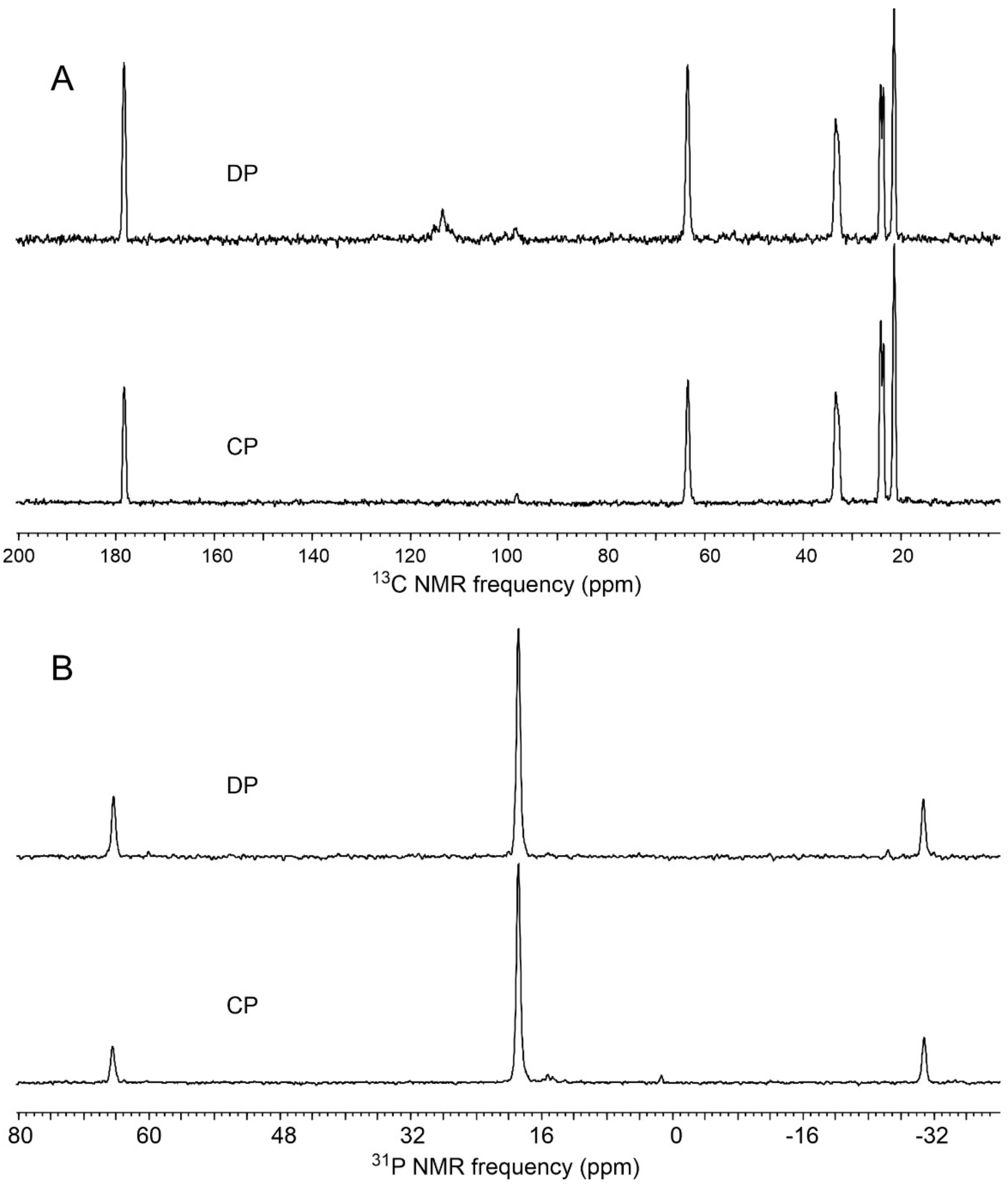

Figure S7: (A) 1D ^{13}C NMR spectra of a 1.25 mg sample of uniformly ^{15}N,^{13}C-labeled L-valine powder, obtained with direct pulsing, four scans, and a 9.0 s recycle delay (top) or with ^{1}H-^{13}C cross-polarization, two scans, and 2.0 s recycle delay (bottom). (B) 1D ^{31}P NMR spectra of a 5.0 mg sample of aminomethyl phosphonic acid (AMPA) powder, obtained with direct pulsing and one scan after a 60 min delay (top) or with ^{1}H-^{31}P cross-polarization, 32 scans, and 40 s recycle delay (bottom). Spectra were recorded at 14.1 T with 12.00 kHz MAS. Signal areas in these spectra of standard samples, with known quantities of ^{13}C and ^{31}P, were used to calibrate areas in 1D spectra of MBPA-FG30/AAO20 samples.

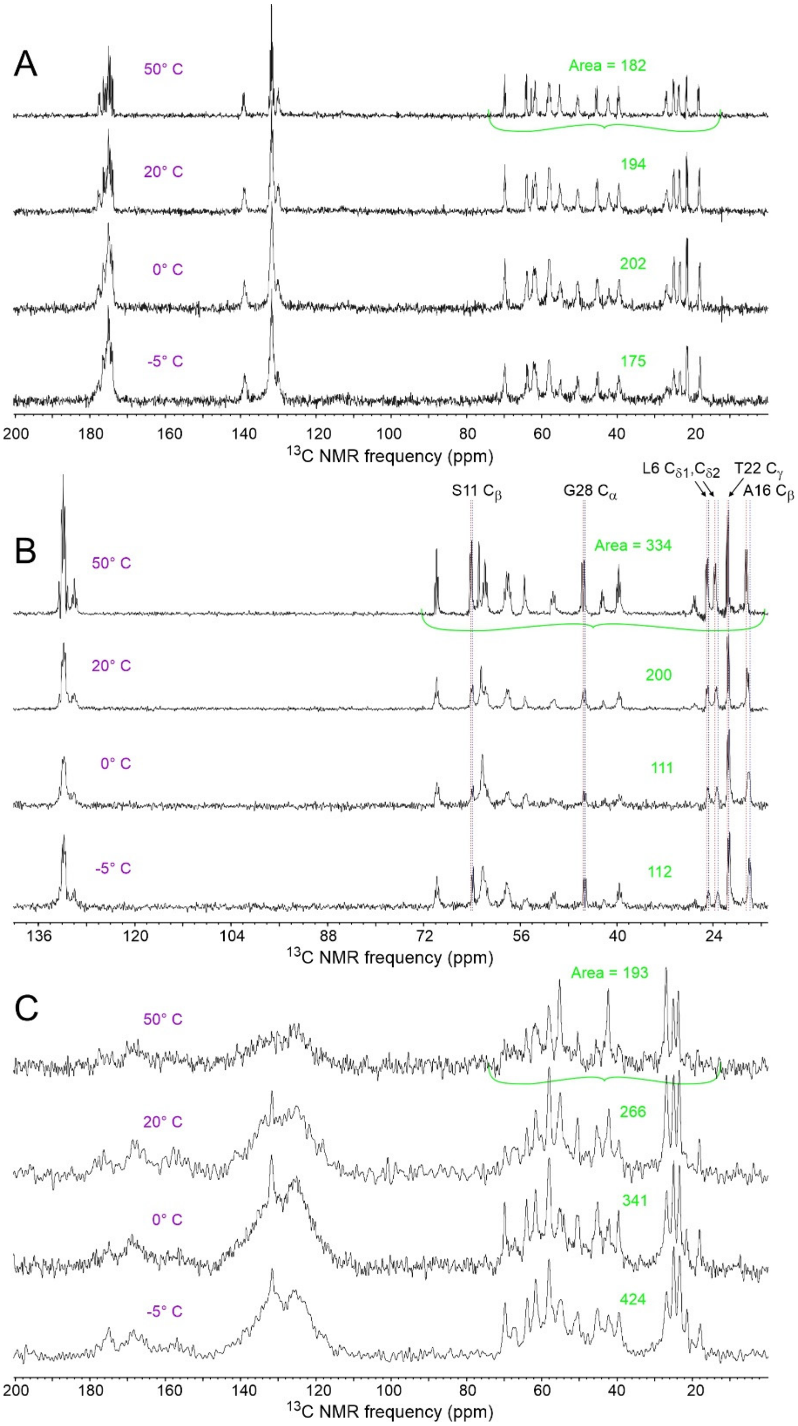

Figure S8: 1D ^{13}C NMR spectra of hydrated MBPA-C-FG30-LSAFTG/AAO20 obtained with direct pulsing and 1024 scans (A), ^{1}H-^{13}C INEPT and 1024 scans (B), or ^{1}H-^{13}C cross-polarization and 5120 scans (C). Spectra were recorded at the indicated sample temperatures. Areas of aliphatic signals are indicated in green. The same units are used for areas in all spectra, making them directly comparable. Uncertainties in these areas are approximately 10%. Peak positions with the strongest temperature dependences are indicated by vertical dashed lines in panel B, with blue and red lines indicating positions at -5° C and 50° C, respectively.

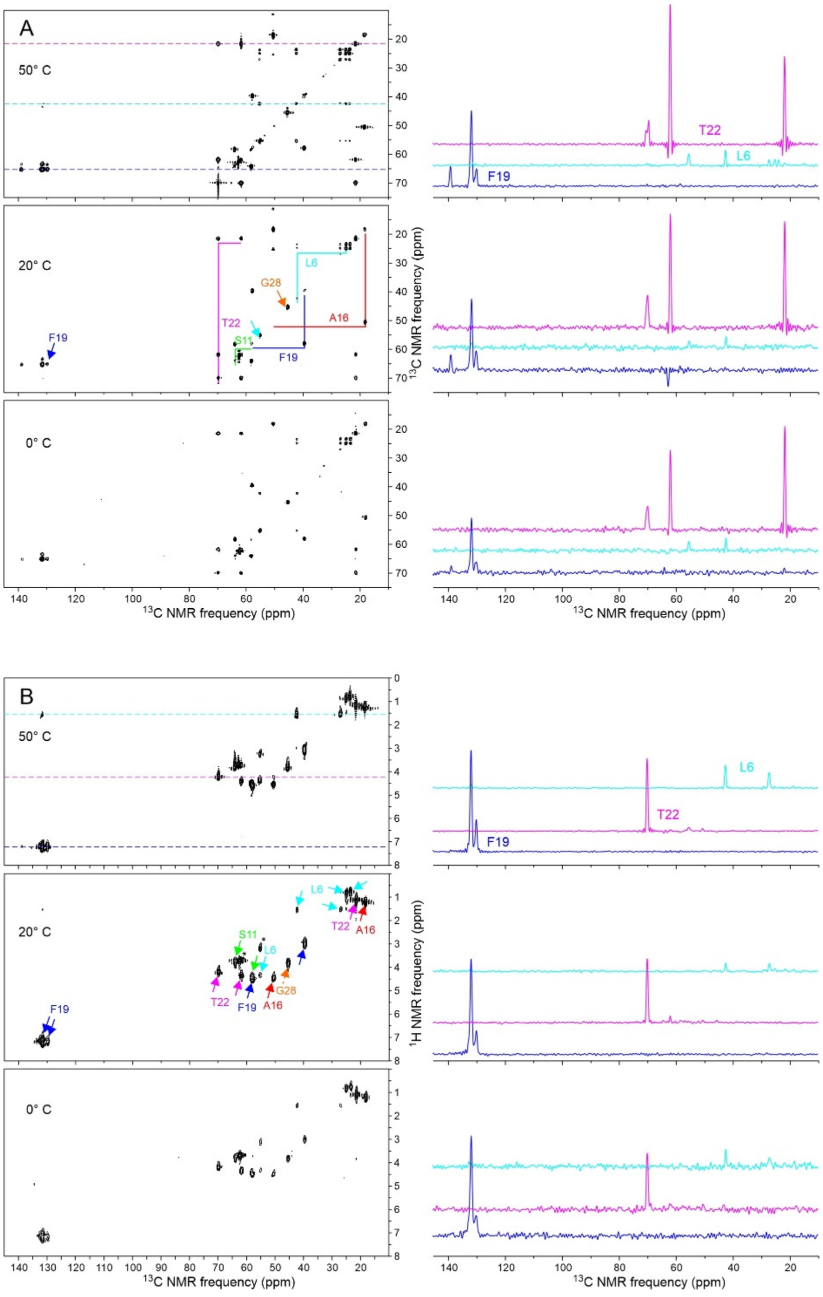

Figure S9: (A) 2D ^{13}C-^{13}C NMR spectra of hydrated MBPA-C-FG30-LSAFTG/AAO20, obtained with ^{1}H-^{13}C INEPT and 12.5 ms isotropic ^{13}C-^{13}C mixing at the indicated sample temperatures. 1D slices, at the positions of color-coded dashed lines the 2D spectra, are shown to the right of each 2D spectrum. Spectra at 0° C, 20° C, and 50° C were recorded with 512, 832, and 864 scans per complex t_1 point, respectively. (B) 2D ^{1}H-^{13}C NMR spectra of hydrated MBPA-C-FG30-LSAFTG/AAO20, obtained with ^{1}H-^{13}C INEPT at the indicated sample temperatures. 1D slices, at the positions of color-coded dashed lines the 2D spectra, are shown to the right of each 2D spectrum. All 2D ^{1}H-^{13}C NMR spectra were recorded with 512 scans per complex t_1 point. Contour levels increase by successive factors of 1.8 in all 2D spectra in panels A and B.

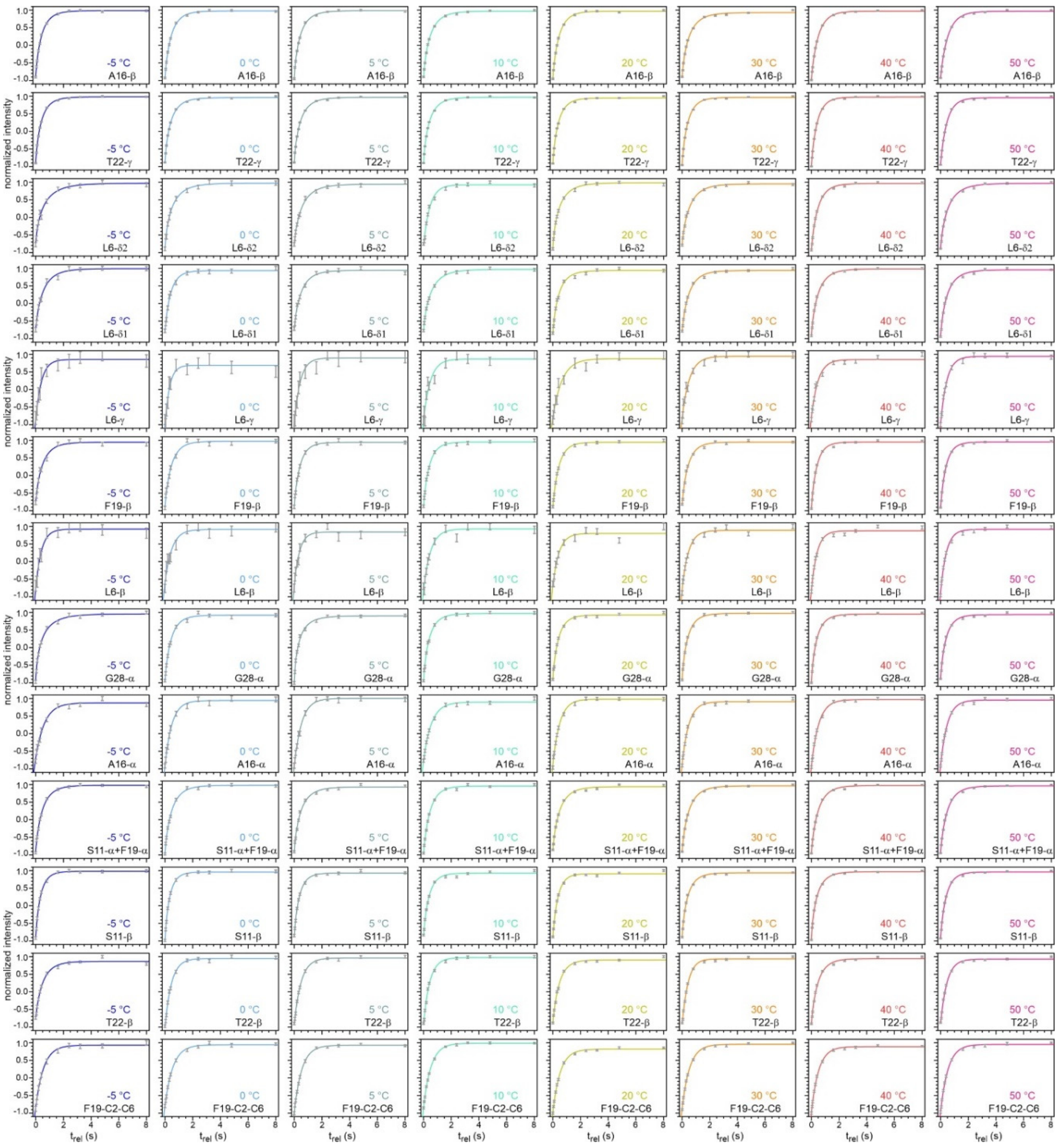

Figure S10: Full set of T_1^H data for hydrated MBPA-C-FG30-LSAFTG/AAO20, obtained with the pulse sequence in Fig. S2H at sample temperatures from -5° C to 50° C. ^{1}H inversion-recovery data were detected through ^{13}C NMR signals of ^{13}C-labeled sites. For example, data labeled T22-γ were detected through signals of the C_γ site of T22. Signals from S11 C_α and F19 C_α were not resolved and were therefore measured as a single set of data. Signals from the C_2-C_6 aromatic sites of F19 are labeled F19-C2-6. Error bars were calculated from the RMS noise in the individual ^{13}C NMR spectra. Solid lines are single-exponential (L6-γ, L6-β, A16-α, F19-C2-6) or stretched-exponential (all others) fits.

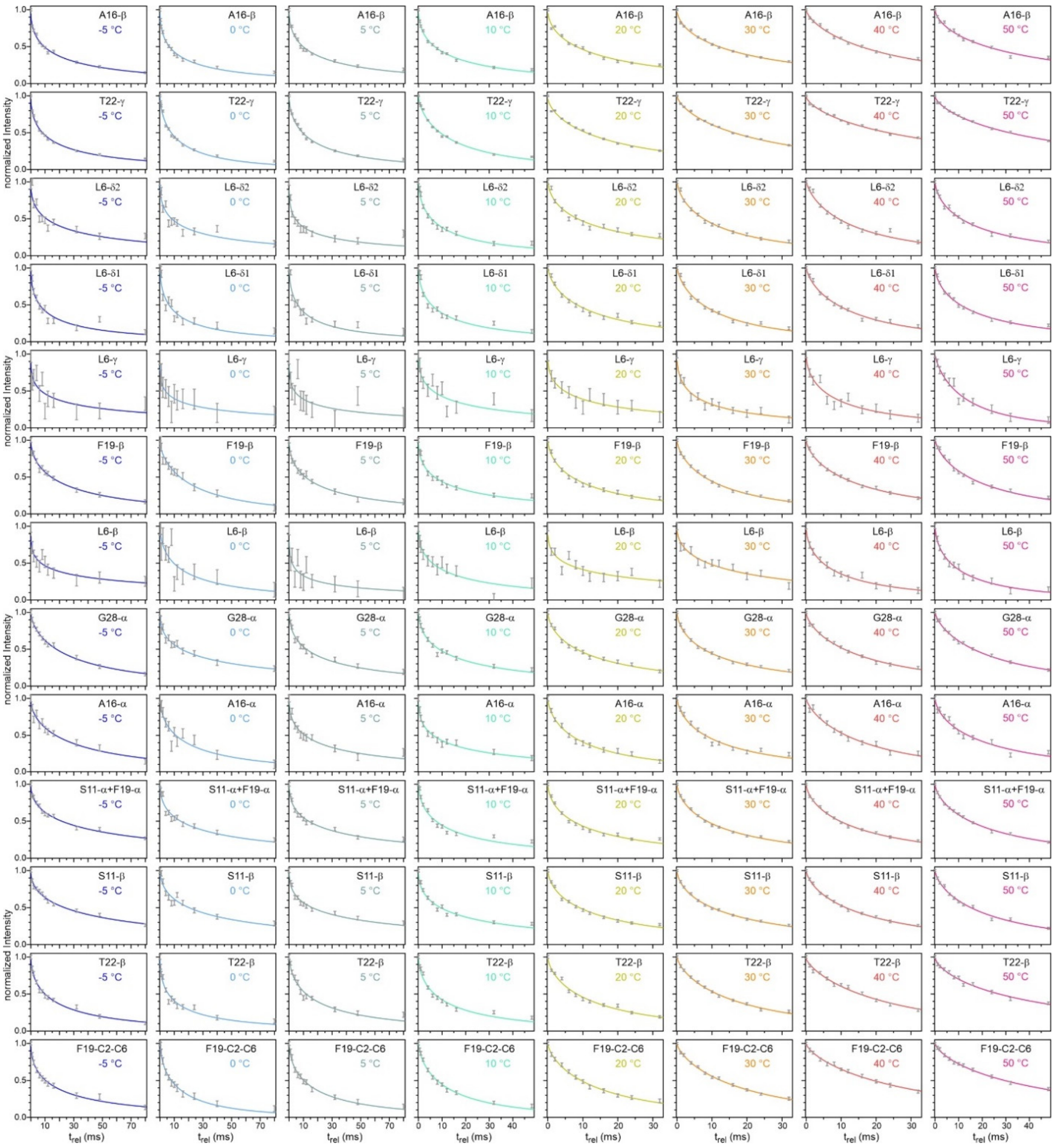

Figure S11: Full set of $T_{1\rho}^H$ data for hydrated MBPA-C-FG30-LSAFTG/AAO20, obtained with the pulse sequence in Fig. S2I and labeled as in Fig. S10. Solid lines are stretched-exponential fits.

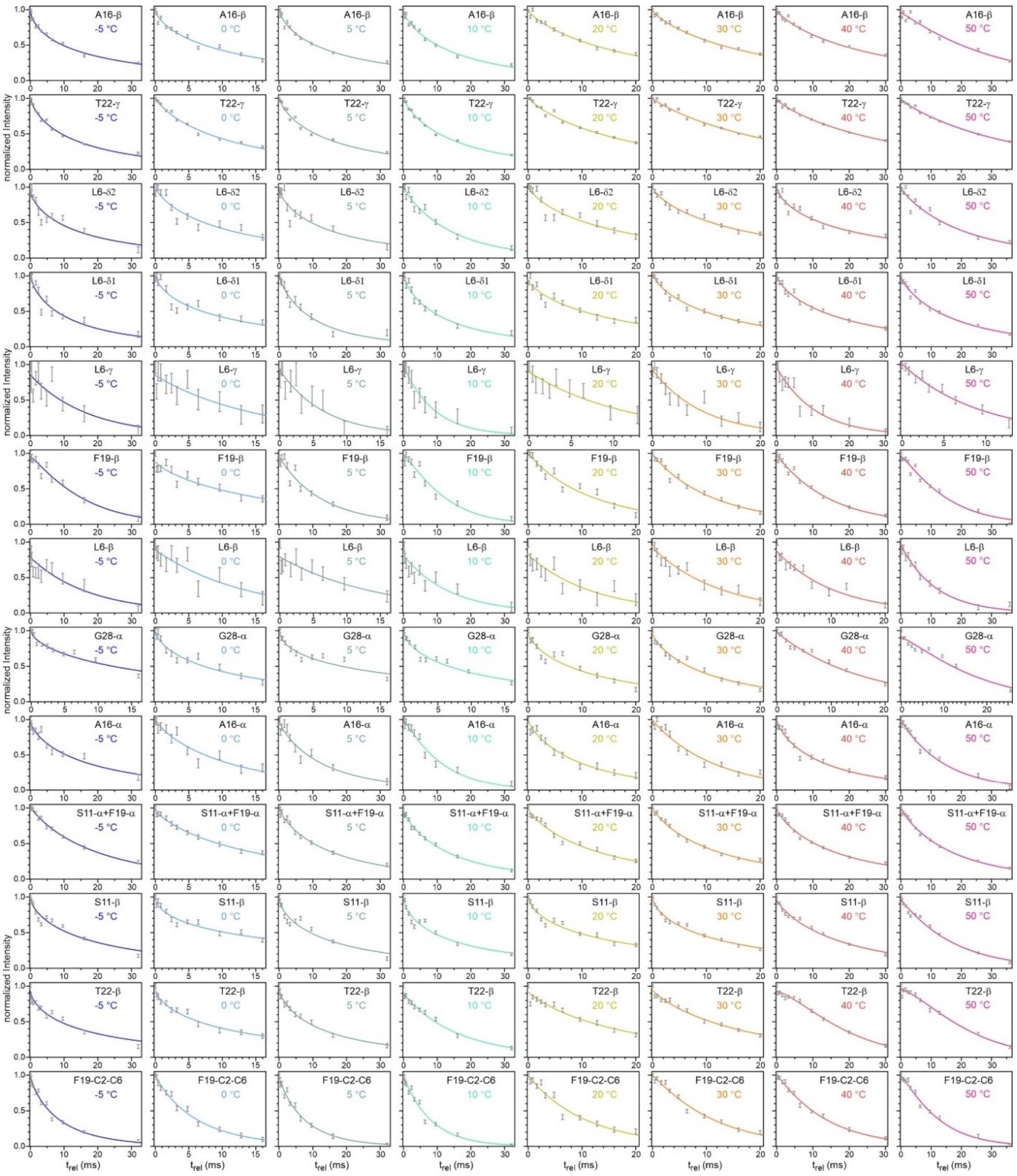

Figure S12: Full set of T_2^H data for hydrated MBPA-C-FG30-LSAFTG/AAO20, obtained with the pulse sequence in Fig. S2J and labeled as in Fig. S10. Solid lines are single-exponential (L6-γ, L6-β) or stretched-exponential (all others) fits.

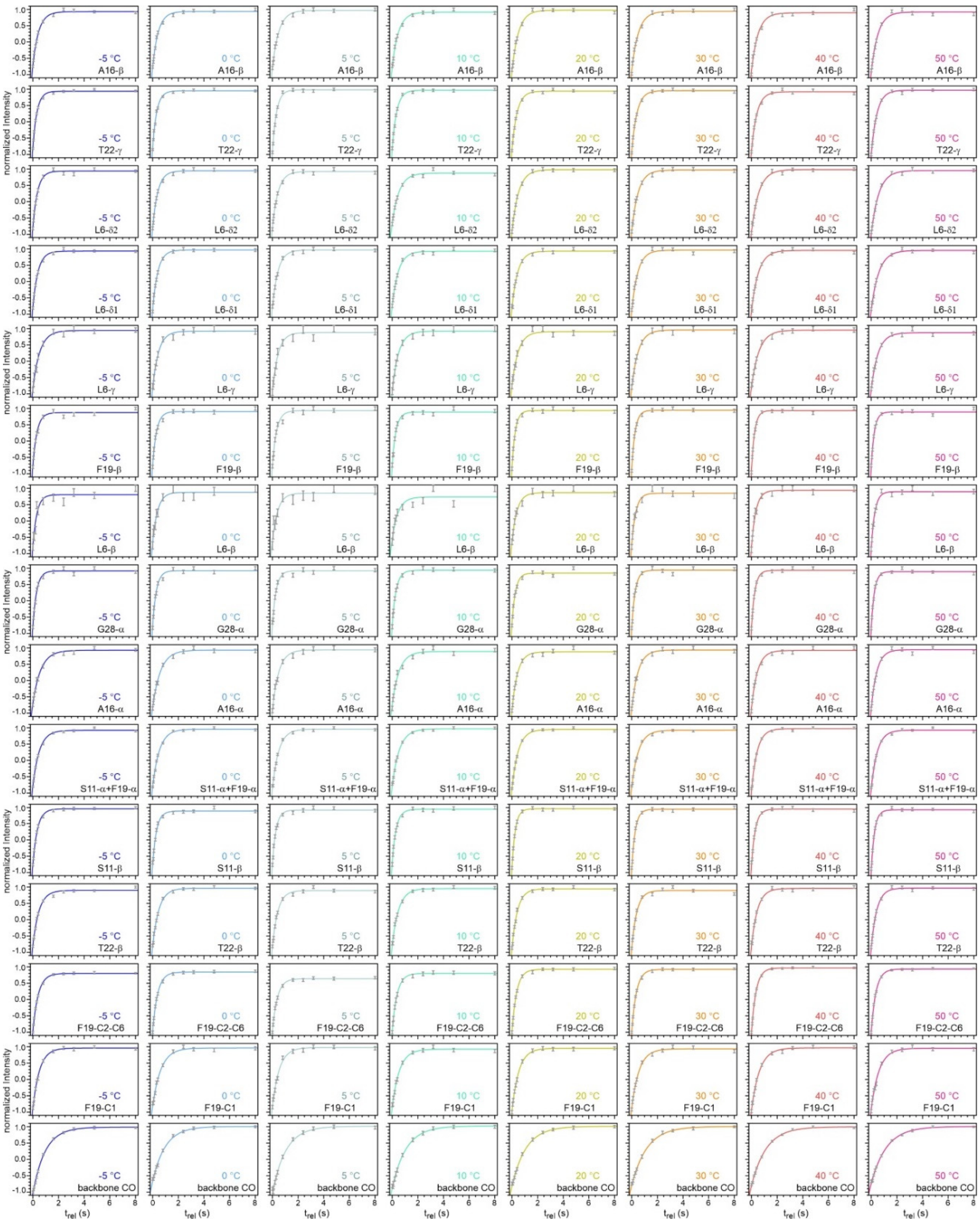

Figure S13: Full set of T_1^C data for hydrated MBPA-C-FG30-LSAFTG/AAO20, obtained with the pulse sequence in Fig. S2K and labeled as in Fig. S10. Solid lines are single-exponential fits.

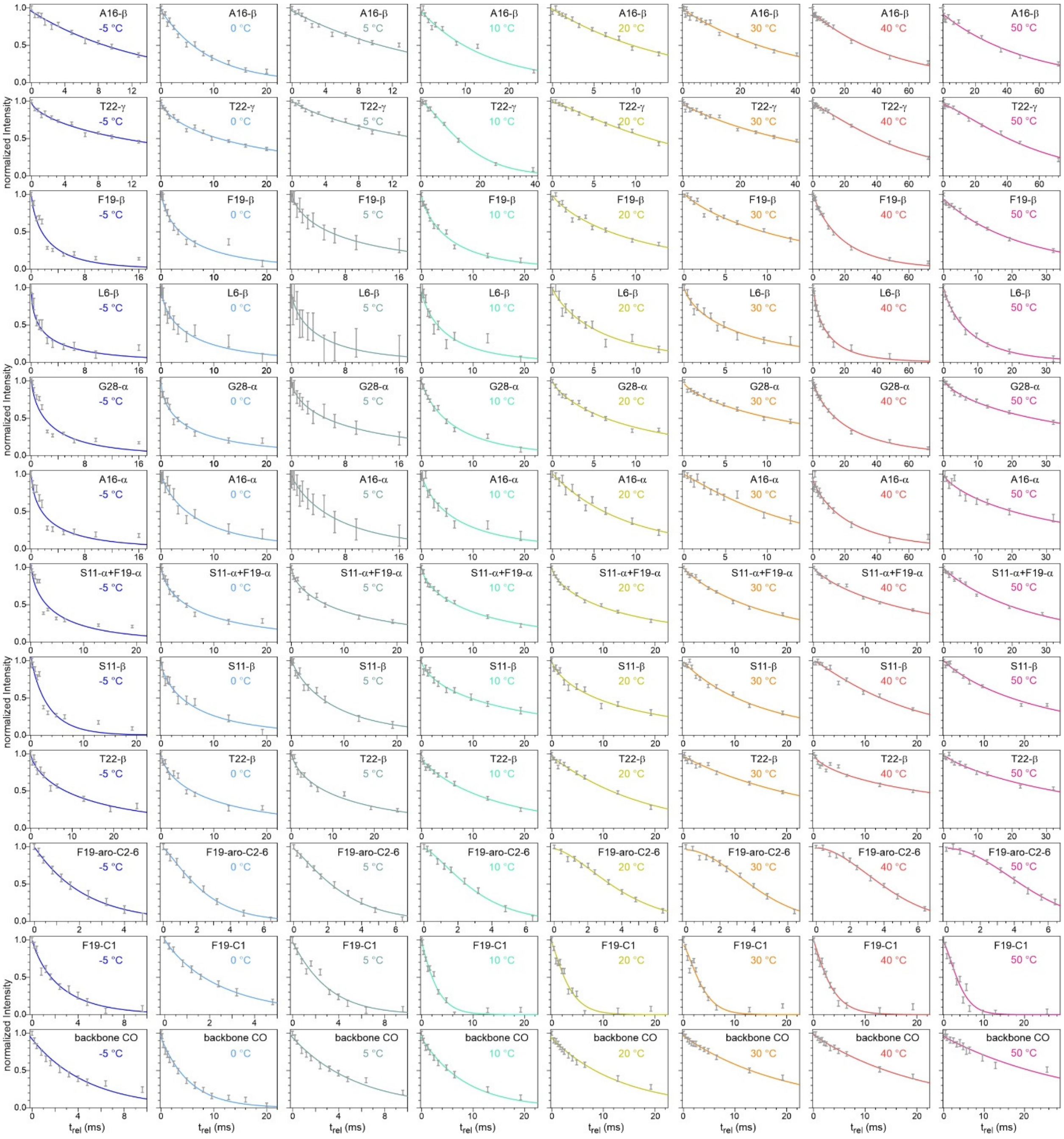

Figure S14: Full set of T_2^C data for hydrated MBPA-C-FG30-LSAFTG/AAO20, obtained with the pulse sequence in Fig. S2L and labeled as in Fig. S10. Solid lines are single-exponential (A16-β, backbone CO) or stretched-exponential (all others) fits.

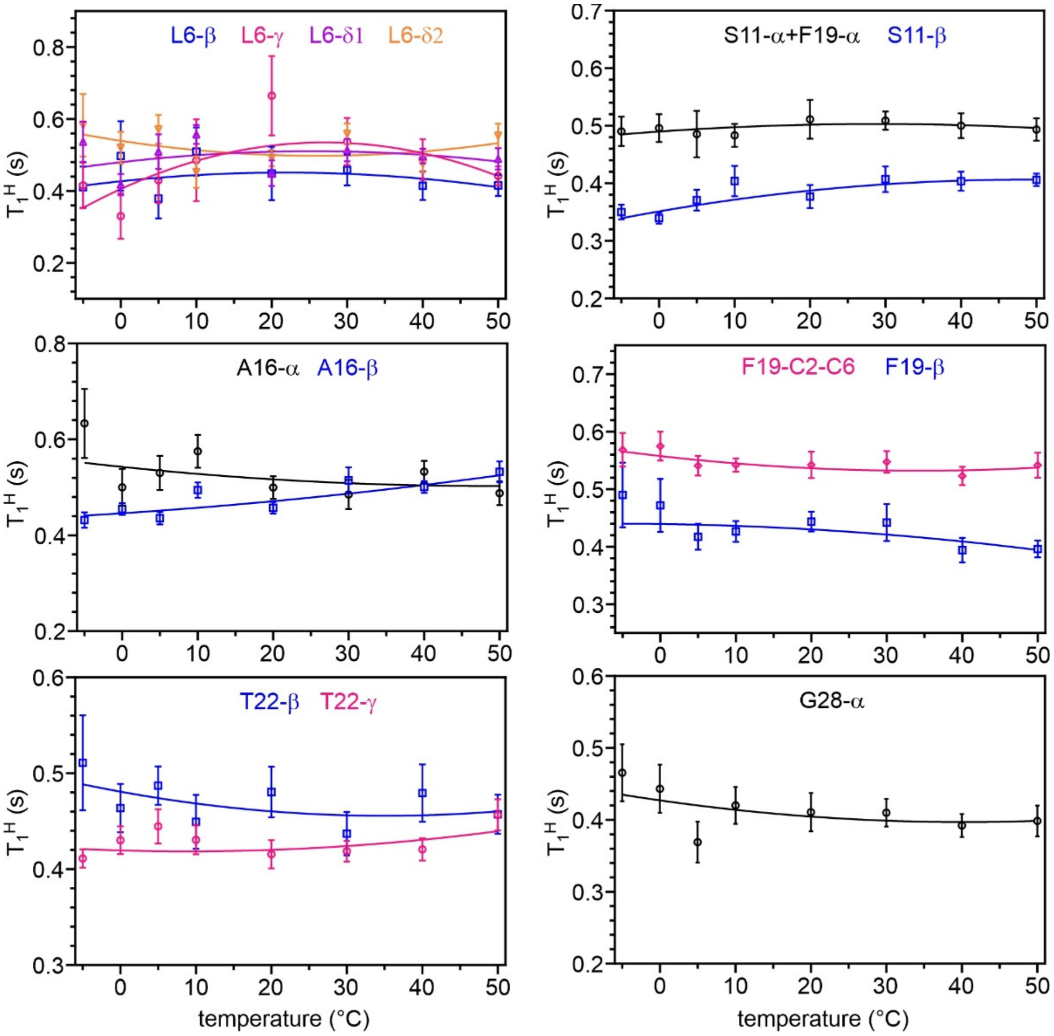

Figure S15: Temperature dependences of T_1^H values determined from data in Fig. S10. Error bars are standard errors reported by the GraphPad fitting software. Solid lines are second-order polynomial functions, serving as guides to the eye.

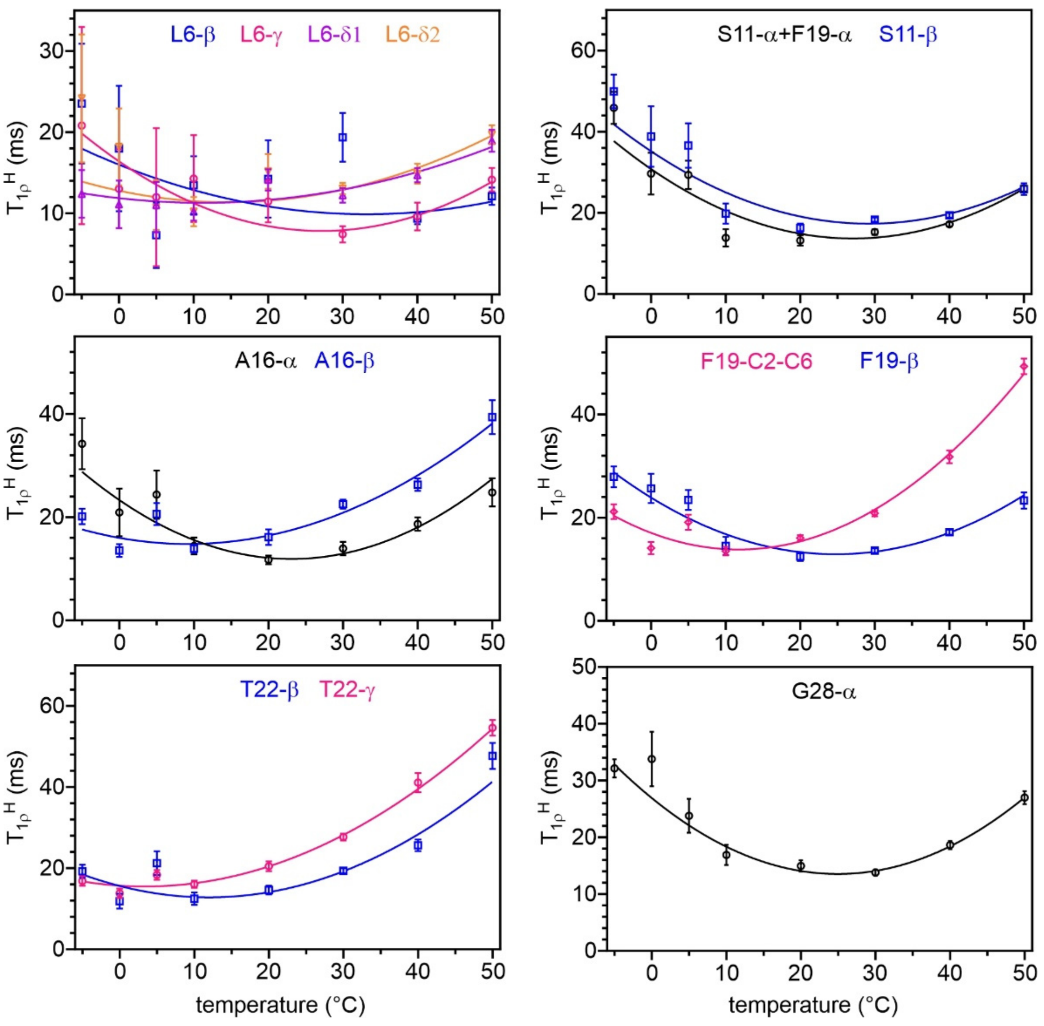

Figure S16: Temperature dependences of $T_{1\rho}^{H}$ values determined from data in Fig. S11. Error bars are standard errors reported by the GraphPad fitting software. Solid lines are second-order polynomial functions, serving as guides to the eye.

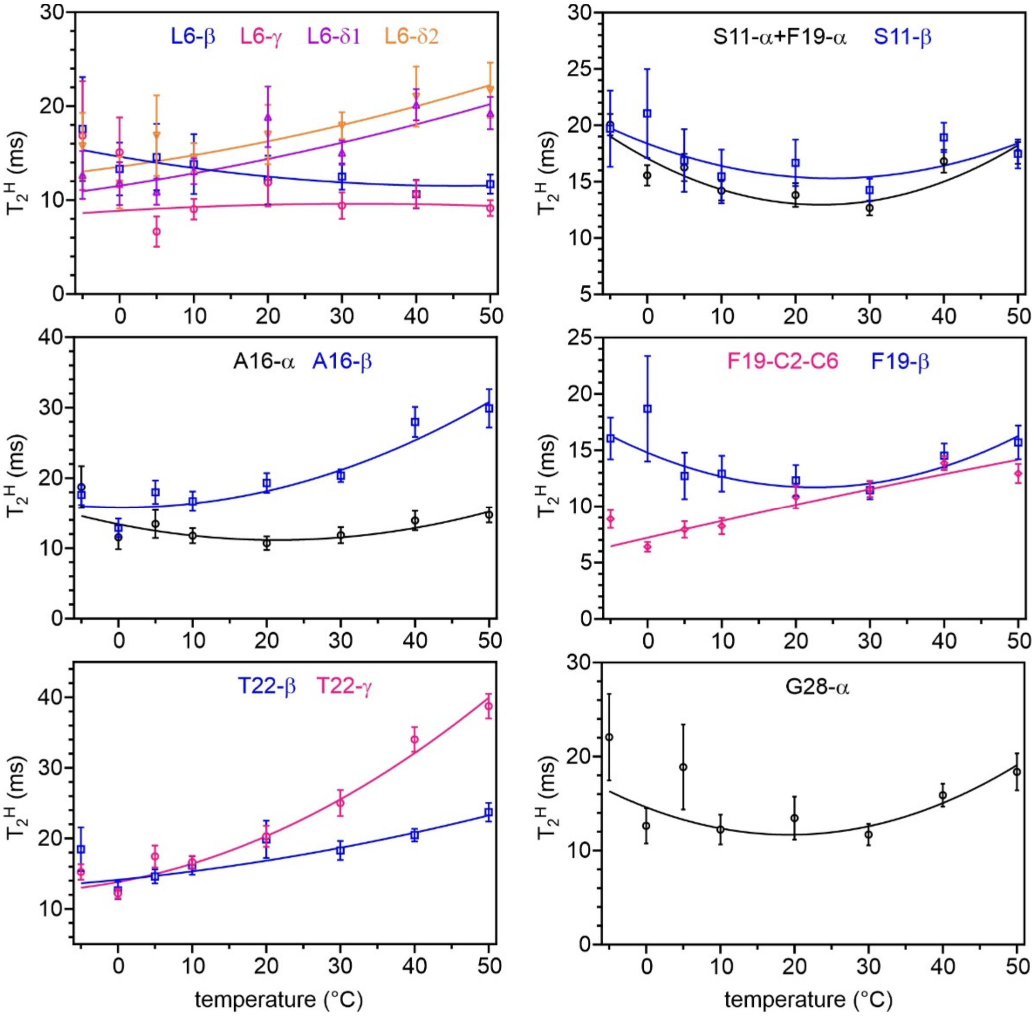

Figure S17: Temperature dependences of T_2^H values determined from data in Fig. S12. Error bars are standard errors reported by the GraphPad fitting software. Solid lines are second-order polynomial functions, serving as guides to the eye.

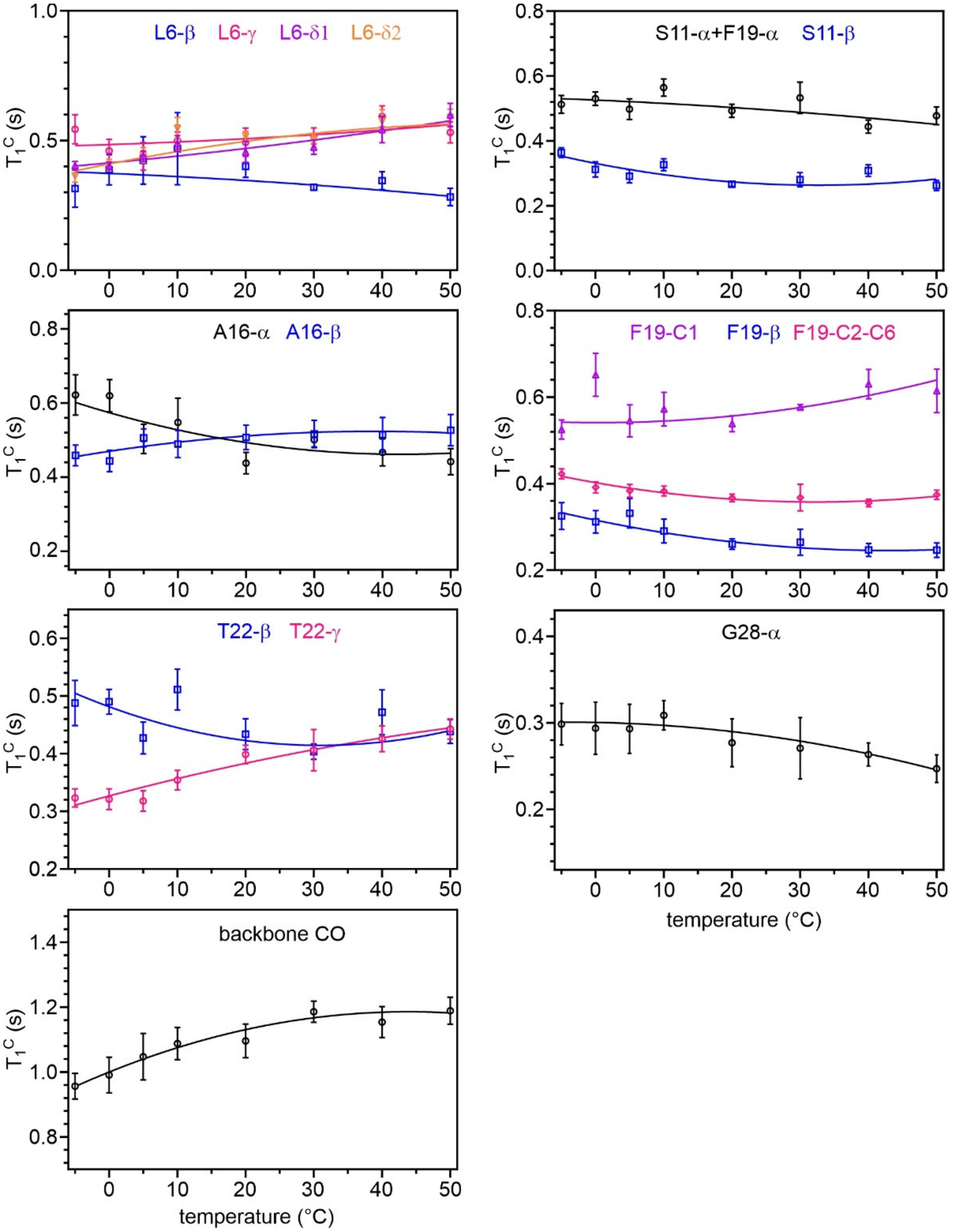

Figure S18: Temperature dependences of T_1^C values determined from data in Fig. S13. Error bars are standard errors reported by the GraphPad fitting software. Solid lines are second-order polynomial functions, serving as guides to the eye.

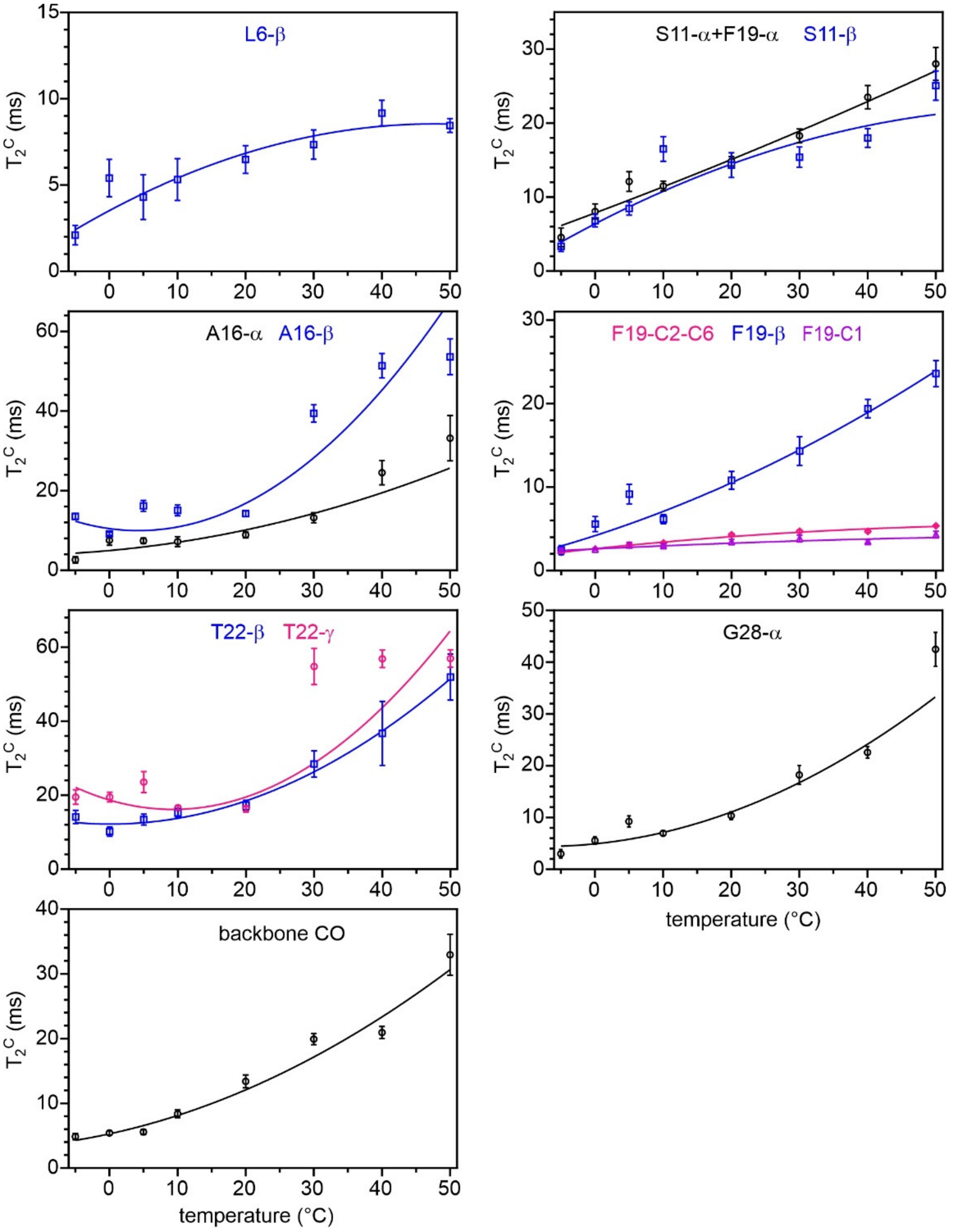

Figure S19: Temperature dependences of T_2^C values determined from data in Fig. S14. Error bars are standard errors reported by the GraphPad fitting software. Solid lines are second-order polynomial functions, serving as guides to the eye.

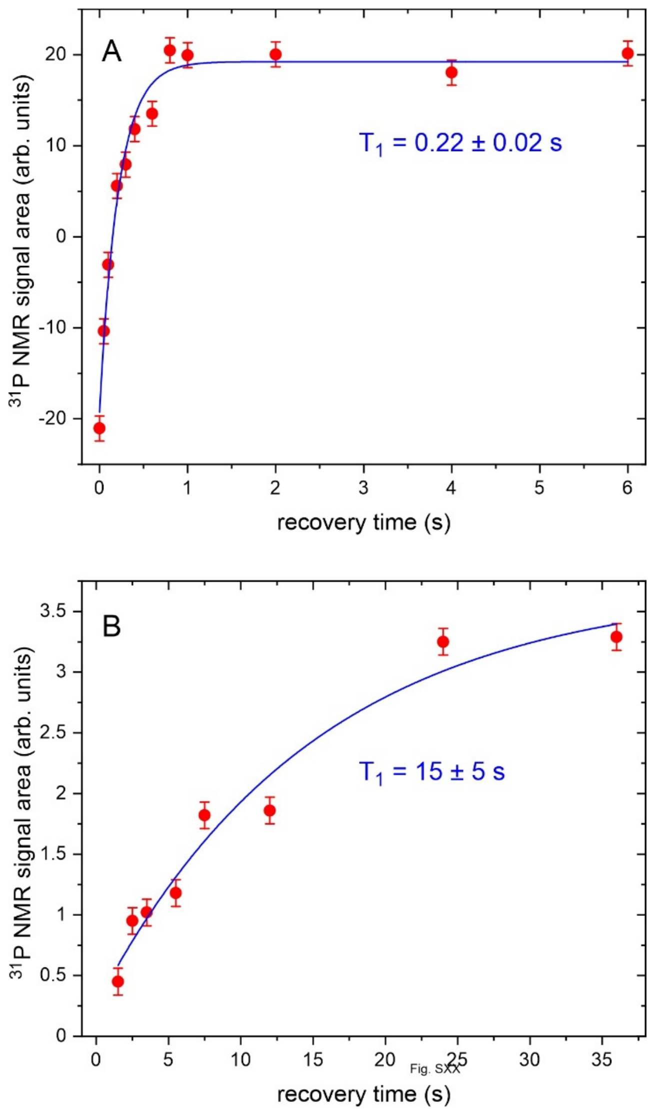

Figure S20: Measurements of ^{31}P spin-lattice relaxation times T_1 in aggregated MBPA-C-FG30-LSAFTG (A) and hydrated MBPA-C-FG30-LSAFTG/AAO20 (B), using inversion-recovery and saturation-recovery methods, respectively. Error bars are uncertainties in signal areas due to noise in the ^{31}P NMR spectra. Blue lines are least-squares fits with single-exponential functions, yielding the indicated values of T_1.

Table S1: T_1^H relaxation time values (seconds) at sample temperatures from -5° C to 50° C, determined from single-exponential or stretched-exponential fits to the data in Fig. S10 and plotted in Fig. S15. For stretched-exponential fits, stretching exponents are given in parentheses. Identical values are listed for the S11 C_α and F19 C_α sites because signals from these sites were not resolved. Uncertainties are standard errors from the fits.

residue and carbon site		-5 °C	0 °C	5 °C	10 °C	20 °C	30 °C	40 °C	50 °C
L6	β	0.41 ± 0.06	0.50 ± 0.10	0.38 ± 0.06	0.51 ± 0.07	0.45 ± 0.07	0.46 ± 0.04	0.41 ± 0.04	0.42 ± 0.03
	γ	0.42 ± 0.06	0.33 ± 0.06	0.43 ± 0.06	0.49 ± 0.11	0.66 ± 0.11	0.54 ± 0.07	0.49 ± 0.06	0.44 ± 0.03
	δ_1	0.54 ± 0.06 (0.83 ± 0.09)	0.42 ± 0.03 (0.92 ± 0.08)	0.51 ± 0.05 (0.86 ± 0.09)	0.56 ± 0.03 (0.82 ± 0.04)	0.45 ± 0.04 (0.86 ± 0.08)	0.51 ± 0.03 (0.88 ± 0.06)	0.50 ± 0.02 (0.85 ± 0.04)	0.49 ± 0.03 (0.83 ± 0.05)
	δ_2	0.58 ± 0.09 (0.75 ± 0.10)	0.52 ± 0.04 (0.78 ± 0.06)	0.57 ± 0.04 (0.82 ± 0.06)	0.45 ± 0.04 (0.98 ± 0.13)	0.49 ± 0.02 (0.79 ± 0.04)	0.56 ± 0.03 (0.84 ± 0.05)	0.48 ± 0.02 (0.90 ± 0.05)	0.55 ± 0.03 (0.81 ± 0.05)
S11	α	0.49 ± 0.03 (0.86 ± 0.05)	0.50 ± 0.02 (0.86 ± 0.05)	0.49 ± 0.04 (0.83 ± 0.07)	0.48 ± 0.02 (0.93 ± 0.05)	0.51 ± 0.03 (0.86 ± 0.06)	0.51 ± 0.02 (0.86 ± 0.03)	0.50 ± 0.02 (0.88 ± 0.04)	0.49 ± 0.02 (0.87 ± 0.04)
	β	0.35 ± 0.01 (0.87 ± 0.04)	0.34 ± 0.01 (0.89 ± 0.03)	0.37 ± 0.02 (0.90 ± 0.06)	0.40 ± 0.03 (0.91 ± 0.08)	0.38 ± 0.02 (0.95 ± 0.07)	0.41 ± 0.02 (0.85 ± 0.05)	0.40 ± 0.02 (0.82 ± 0.04)	0.41 ± 0.01 (0.90 ± 0.03)
A16	α	0.63 ± 0.07	0.50 ± 0.04	0.53 ± 0.04	0.57 ± 0.03	0.50 ± 0.02	0.49 ± 0.03	0.53 ± 0.02	0.49 ± 0.02
	β	0.43 ± 0.02 (0.86 ± 0.04)	0.45 ± 0.01 (0.87 ± 0.03)	0.44 ± 0.01 (0.82 ± 0.03)	0.49 ± 0.02 (0.84 ± 0.03)	0.46 ± 0.01 (0.89 ± 0.03)	0.51 ± 0.03 (0.83 ± 0.05)	0.50 ± 0.01 (0.92 ± 0.03)	0.53 ± 0.02 (0.92 ± 0.04)
F19	α	0.49 ± 0.03 (0.86 ± 0.05)	0.50 ± 0.02 (0.86 ± 0.05)	0.49 ± 0.04 (0.83 ± 0.07)	0.48 ± 0.02 (0.93 ± 0.05)	0.51 ± 0.03 (0.86 ± 0.06)	0.51 ± 0.02 (0.86 ± 0.03)	0.50 ± 0.02 (0.88 ± 0.04)	0.49 ± 0.02 (0.87 ± 0.04)
	β	0.49 ± 0.06 (0.86 ± 0.11)	0.47 ± 0.05 (0.91 ± 0.11)	0.42 ± 0.02 (0.92 ± 0.06)	0.43 ± 0.02 (0.92 ± 0.05)	0.44 ± 0.02 (0.95 ± 0.05)	0.44 ± 0.03 (0.90 ± 0.08)	0.39 ± 0.02 (0.92 ± 0.06)	0.40 ± 0.01 (0.93 ± 0.04)
	C2-C6	0.57 ± 0.03	0.57 ± 0.03	0.54 ± 0.02	0.54 ± 0.01	0.54 ± 0.02	0.55 ± 0.02	0.52 ± 0.02	0.54 ± 0.02
T22	β	0.51 ± 0.05 (0.88 ± 0.10)	0.46 ± 0.03 (1.00 ± 0.07)	0.49 ± 0.02 (0.98 ± 0.05)	0.45 ± 0.03 (0.98 ± 0.08)	0.48 ± 0.03 (0.96 ± 0.07)	0.44 ± 0.02 (1.05 ± 0.08)	0.48 ± 0.03 (0.89 ± 0.07)	0.46 ± 0.02 (1.00 ± 0.06)
	γ	0.41 ± 0.01 (0.84 ± 0.02)	0.43 ± 0.01 (0.82 ± 0.03)	0.44 ± 0.02 (0.81 ± 0.04)	0.43 ± 0.02 (0.82 ± 0.03)	0.42 ± 0.01 (0.87 ± 0.04)	0.42 ± 0.01 (0.88 ± 0.03)	0.42 ± 0.01 (0.91 ± 0.03)	0.46 ± 0.02 (0.94 ± 0.04)
G28	α	0.47 ± 0.04 (0.70 ± 0.05)	0.44 ± 0.03 (0.89 ± 0.08)	0.37 ± 0.03 (0.73 ± 0.06)	0.42 ± 0.03 (0.86 ± 0.06)	0.41 ± 0.03 (0.90 ± 0.07)	0.41 ± 0.02 (0.86 ± 0.05)	0.39 ± 0.02 (0.85 ± 0.04)	0.40 ± 0.02 (0.87 ± 0.06)

Table S2: $T_{1\rho}^{H}$ relaxation time values (milliseconds) at sample temperatures from -5° C to 50° C, determined from stretched-exponential fits to the data in Fig. S11 and plotted in Fig. S16. Stretching exponents are given in parentheses. Identical values are listed for the S11 C_α and F19 C_α sites because signals from these sites were not resolved. Uncertainties are standard errors from the fits.

residue and carbon site		-5 °C	0 °C	5 °C	10 °C	20 °C	30 °C	40 °C	50 °C
L6	β	23.5 ± 7.4 (0.31 ± 0.06)	18.0 ± 7.7 (0.52 ± 0.19)	7.3 ± 4.0 (0.31 ± 0.10)	13.5 ± 3.6 (0.47 ± 0.10)	14.2 ± 4.8 (0.36 ± 0.08)	19.4 ± 3.0 (0.50 ± 0.08)	9.4 ± 0.7 (0.56 ± 0.04)	12.1 ± 1.1 (0.59 ± 0.05)
	γ	20.8 ± 12.2 (0.34 ± 0.12)	13.0 ± 4.9 (0.30 ± 0.06)	12.0 ± 8.5 (0.31 ± 0.13)	14.3 ± 5.4 (0.42 ± 0.12)	11.5 ± 2.6 (0.42 ± 0.07)	7.4 ± 1.0 (0.46 ± 0.05)	9.6 ± 1.7 (0.56 ± 0.10)	14.2 ± 1.4 (0.74 ± 0.09)
	δ_1	12.4 ± 2.9 (0.46 ± 0.08)	11.1 ± 2.9 (0.49 ± 0.10)	11.0 ± 3.1 (0.48 ± 0.10)	10.2 ± 1.8 (0.50 ± 0.07)	14.1 ± 1.3 (0.61 ± 0.06)	12.2 ± 0.9 (0.67 ± 0.06)	14.7 ± 0.9 (0.69 ± 0.05)	18.9 ± 1.3 (0.60 ± 0.04)
	δ_2	24.2 ± 7.9 (0.44 ± 0.10)	18.2 ± 4.7 (0.42 ± 0.08)	10.7 ± 3.1 (0.36 ± 0.06)	10.2 ± 1.1 (0.53 ± 0.05)	15.2 ± 2.1 (0.54 ± 0.07)	13.1 ± 0.7 (0.64 ± 0.04)	14.9 ± 1.2 (0.70 ± 0.07)	19.7 ± 1.2 (0.62 ± 0.04)
S11	α	45.9 ± 4.0 (0.48 ± 0.03)	29.7 ± 5.1 (0.44 ± 0.06)	29.4 ± 3.5 (0.44 ± 0.04)	13.8 ± 2.1 (0.50 ± 0.07)	13.2 ± 1.3 (0.54 ± 0.05)	15.3 ± 0.7 (0.61 ± 0.03)	17.2 ± 0.6 (0.65 ± 0.03)	26.0 ± 0.7 (0.67 ± 0.02)
	β	50.0 ± 4.2 (0.50 ± 0.03)	38.8 ± 7.5 (0.47 ± 0.07)	36.6 ± 5.5 (0.40 ± 0.04)	19.8 ± 2.5 (0.45 ± 0.05)	16.3 ± 1.1 (0.57 ± 0.04)	18.3 ± 0.5 (0.60 ± 0.02)	19.4 ± 0.6 (0.67 ± 0.03)	25.9 ± 1.4 (0.68 ± 0.04)
A16	α	34.2 ± 5.0 (0.59 ± 0.08)	20.9 ± 4.6 (0.54 ± 0.11)	24.4 ± 4.7 (0.45 ± 0.06)	14.4 ± 1.7 (0.41 ± 0.04)	11.7 ± 0.8 (0.61 ± 0.04)	13.9 ± 1.3 (0.61 ± 0.06)	18.7 ± 1.3 (0.75 ± 0.07)	24.8 ± 2.7 (0.64 ± 0.08)
	β	20.1 ± 1.5 (0.47 ± 0.03)	13.5 ± 1.2 (0.46 ± 0.03)	20.6 ± 2.1 (0.47 ± 0.04)	13.9 ± 0.8 (0.50 ± 0.02)	16.1 ± 1.5 (0.56 ± 0.05)	22.5 ± 0.9 (0.59 ± 0.03)	26.3 ± 1.2 (0.71 ± 0.04)	39.4 ± 3.3 (0.63 ± 0.06)
F19	α	45.9 ± 4.0 (0.48 ± 0.03)	29.7 ± 5.1 (0.44 ± 0.06)	29.4 ± 3.5 (0.44 ± 0.04)	13.8 ± 2.1 (0.50 ± 0.07)	13.2 ± 1.3 (0.54 ± 0.05)	15.3 ± 0.7 (0.61 ± 0.03)	17.2 ± 0.6 (0.65 ± 0.03)	26.0 ± 0.7 (0.67 ± 0.02)
	β	27.9 ± 2.0 (0.56 ± 0.03)	25.7 ± 2.8 (0.66 ± 0.08)	23.4 ± 1.9 (0.52 ± 0.04)	14.5 ± 1.7 (0.45 ± 0.04)	12.4 ± 0.8 (0.57 ± 0.04)	13.6 ± 0.5 (0.67 ± 0.03)	17.2 ± 0.5 (0.67 ± 0.02)	23.3 ± 1.6 (0.69 ± 0.05)
	C2-C6	21.1 ± 1.4 (0.50 ± 0.03)	14.1 ± 1.2 (0.59 ± 0.05)	19.1 ± 1.5 (0.56 ± 0.04)	13.5 ± 0.8 (0.63 ± 0.04)	16.1 ± 0.6 (0.70 ± 0.03)	20.8 ± 0.6 (0.75 ± 0.03)	31.8 ± 1.2 (0.75 ± 0.04)	49.3 ± 1.5 (0.71 ± 0.02)
T22	β	19.2 ± 1.6 (0.53 ± 0.04)	11.9 ± 1.8 (0.47 ± 0.05)	21.2 ± 3.0 (0.54 ± 0.06)	12.5 ± 1.5 (0.54 ± 0.06)	14.6 ± 1.1 (0.65 ± 0.05)	19.4 ± 0.6 (0.71 ± 0.03)	25.6 ± 1.5 (0.77 ± 0.06)	47.7 ± 3.2 (0.67 ± 0.05)
	γ	16.8 ± 1.2 (0.48 ± 0.03)	13.8 ± 1.2 (0.57 ± 0.04)	18.3 ± 1.2 (0.55 ± 0.03)	16.0 ± 0.9 (0.64 ± 0.04)	20.5 ± 1.2 (0.64 ± 0.05)	27.7 ± 0.8 (0.69 ± 0.03)	41.1 ± 2.4 (0.68 ± 0.04)	54.6 ± 2.0 (0.69 ± 0.03)
G28	α	32.1 ± 1.6 (0.63 ± 0.03)	33.8 ± 4.8 (0.44 ± 0.05)	23.8 ± 3.0 (0.48 ± 0.05)	16.9 ± 1.8 (0.51 ± 0.05)	15.0 ± 1.0 (0.59 ± 0.04)	13.8 ± 0.5 (0.60 ± 0.02)	18.7 ± 0.7 (0.70 ± 0.04)	27.0 ± 1.1 (0.71 ± 0.04)

Table S3: T_2^H relaxation time values (milliseconds) at sample temperatures from -5° C to 50° C, determined from single-exponential or stretched-exponential fits to the data in Fig. S12 and plotted in Fig. S17. For stretched-exponential fits, stretching exponents are given in parentheses. Identical values are listed for the S11 C_α and F19 C_α sites because signals from these sites were not resolved. Uncertainties are standard errors from the fits.

residue and carbon site		-5 °C	0 °C	5 °C	10 °C	20 °C	30 °C	40 °C	50 °C
L6	β	17.6 ± 5.5	13.3 ± 2.8	14.6 ± 3.6	13.8 ± 3.2	12.1 ± 2.6	12.5 ± 1.4	10.6 ± 1.5	11.7 ± 1.0
	γ	16.9 ± 5.8	15.1 ± 3.7	6.7 ± 1.6	9.0 ± 1.1	11.9 ± 2.5	9.4 ± 1.4	10.6 ± 1.5	9.2 ± 0.8
	δ_1	12.7 ± 2.5 (0.7 ± 0.16)	11.9 ± 2.7 (0.61 ± 0.17)	10.9 ± 1.4 (0.78 ± 0.13)	13.1 ± 1.4 (0.72 ± 0.09)	18.9 ± 3.2 (0.73 ± 0.17)	15.1 ± 1.3 (0.73 ± 0.08)	20.2 ± 1.7 (0.74 ± 0.08)	19.3 ± 1.7 (0.80 ± 0.09)
	δ_2	15.7 ± 3.6 (0.68 ± 0.16)	11.8 ± 2.3 (0.73 ± 0.19)	16.9 ± 4.3 (0.69 ± 0.21)	14.6 ± 1.5 (0.93 ± 0.14)	17.0 ± 3.2 (0.72 ± 0.18)	17.9 ± 1.4 (0.75 ± 0.09)	21.0 ± 3.2 (0.66 ± 0.12)	21.7 ± 2.9 (0.89 ± 0.17)
S11	α	20.1 ± 1.0 (0.89 ± 0.05)	15.6 ± 0.9 (0.81 ± 0.07)	16.3 ± 1.2 (0.79 ± 0.07)	14.2 ± 0.9 (0.83 ± 0.07)	13.8 ± 1.1 (0.82 ± 0.09)	12.7 ± 0.7 (0.82 ± 0.06)	16.8 ± 1.0 (0.80 ± 0.07)	17.6 ± 1.0 (0.89 ± 0.06)
	β	19.7 ± 3.4 (0.66 ± 0.12)	21.1 ± 3.9 (0.53 ± 0.10)	16.9 ± 2.8 (0.66 ± 0.12)	15.5 ± 2.4 (0.61 ± 0.10)	16.7 ± 2.1 (0.60 ± 0.08)	14.3 ± 1.0 (0.65 ± 0.05)	18.9 ± 1.3 (0.82 ± 0.08)	17.5 ± 1.3 (1.05 ± 0.11)
A16	α	18.7 ± 3.0 (0.7 ± 0.12)	11.6 ± 1.7 (0.83 ± 0.18)	13.4 ± 2.0 (0.84 ± 0.17)	11.8 ± 1.1 (1.08 ± 0.16)	10.7 ± 1.0 (0.81 ± 0.10)	11.9 ± 1.2 (0.98 ± 0.16)	13.9 ± 1.4 (0.75 ± 0.09)	14.8 ± 1.1 (0.98 ± 0.10)
	β	17.6 ± 1.4 (0.61 ± 0.05)	12.9 ± 1.3 (0.69 ± 0.09)	18.0 ± 1.7 (0.66 ± 0.07)	16.7 ± 1.4 (0.76 ± 0.08)	19.3 ± 1.4 (0.83 ± 0.09)	20.4 ± 0.9 (0.82 ± 0.06)	28.0 ± 2.1 (0.81 ± 0.09)	29.9 ± 2.7 (1.07 ± 0.17)
F19	α	20.1 ± 1.0 (0.89 ± 0.05)	15.6 ± 0.9 (0.81 ± 0.07)	16.3 ± 1.2 (0.79 ± 0.07)	14.2 ± 0.9 (0.83 ± 0.07)	13.8 ± 1.1 (0.82 ± 0.09)	12.7 ± 0.7 (0.82 ± 0.06)	16.8 ± 1.0 (0.80 ± 0.07)	17.6 ± 1.0 (0.89 ± 0.06)
	β	16.1 ± 1.9 (1.14 ± 0.2)	18.7 ± 4.7 (0.78 ± 0.26)	12.7 ± 2.1 (0.96 ± 0.23)	12.9 ± 1.6 (1.22 ± 0.25)	12.3 ± 1.4 (0.92 ± 0.16)	11.5 ± 0.8 (0.94 ± 0.10)	14.5 ± 1.1 (1.03 ± 0.12)	15.7 ± 1.5 (1.17 ± 0.17)
	C2-C6	8.9 ± 0.8 (0.82 ± 0.09)	6.4 ± 0.4 (0.93 ± 0.09)	7.9 ± 0.7 (0.9 ± 0.11)	8.2 ± 0.7 (1.00 ± 0.13)	10.9 ± 1.0 (0.97 ± 0.14)	11.5 ± 0.7 (1.07 ± 0.11)	13.9 ± 0.6 (1.05 ± 0.07)	12.9 ± 0.8 (1.14 ± 0.12)
T22	β	18.5 ± 3.1 (0.62 ± 0.10)	12.6 ± 1.3 (0.63 ± 0.08)	14.6 ± 1.0 (0.68 ± 0.05)	16.1 ± 1.3 (0.94 ± 0.11)	19.8 ± 2.6 (0.86 ± 0.18)	18.3 ± 1.3 (0.86 ± 0.10)	20.5 ± 0.9 (1.40 ± 0.13)	23.7 ± 1.3 (1.40 ± 0.15)
	γ	15.2 ± 1.1 (0.69 ± 0.05)	12.2 ± 0.7 (0.79 ± 0.07)	17.4 ± 1.6 (0.7 ± 0.08)	16.6 ± 0.9 (0.76 ± 0.05)	20.3 ± 1.5 (0.84 ± 0.09)	25.0 ± 1.9 (0.91 ± 0.10)	34.0 ± 1.8 (0.85 ± 0.06)	38.7 ± 1.8 (0.91 ± 0.06)
G28	α	22.1 ± 4.6 (0.64 ± 0.13)	12.6 ± 1.9 (0.64 ± 0.12)	18.9 ± 4.5 (0.54 ± 0.12)	12.2 ± 1.6 (0.63 ± 0.1)	13.5 ± 2.3 (0.70 ± 0.15)	11.7 ± 1.2 (0.77 ± 0.10)	15.9 ± 1.2 (1.00 ± 0.13)	18.4 ± 2.0 (1.22 ± 0.22)

Table S4: T_1^C relaxation time values (seconds) at sample temperatures from -5° C to 50° C, determined from single-exponential fits to the data in Fig. S13 and plotted in Fig. S18. Identical values are listed for the S11 C_β and F19 C_α sites because signals from these sites were not resolved. A single set of values is given for backbone CO sites, which also did not have resolved signals. Uncertainties are standard errors from the fits.

residue and carbon site		-5 °C	0 °C	5 °C	10 °C	20 °C	30 °C	40 °C	50 °C
L6	β	0.32 ± 0.07	0.39 ± 0.06	0.42 ± 0.09	0.47 ± 0.14	0.40 ± 0.04	0.32 ± 0.01	0.35 ± 0.03	0.28 ± 0.03
	γ	0.54 ± 0.06	0.46 ± 0.04	0.44 ± 0.05	0.50 ± 0.03	0.49 ± 0.05	0.52 ± 0.03	0.59 ± 0.04	0.53 ± 0.04
	δ_1	0.40 ± 0.02	0.40 ± 0.01	0.44 ± 0.02	0.49 ± 0.03	0.46 ± 0.04	0.47 ± 0.03	0.54 ± 0.05	0.60 ± 0.04
	δ_2	0.36 ± 0.02	0.41 ± 0.02	0.44 ± 0.03	0.55 ± 0.04	0.52 ± 0.02	0.50 ± 0.01	0.58 ± 0.04	0.59 ± 0.03
S11	α	0.51 ± 0.03	0.53 ± 0.02	0.50 ± 0.03	0.56 ± 0.03	0.49 ± 0.02	0.53 ± 0.05	0.44 ± 0.02	0.48 ± 0.03
	β	0.36 ± 0.02	0.31 ± 0.02	0.29 ± 0.02	0.33 ± 0.02	0.27 ± 0.01	0.28 ± 0.02	0.31 ± 0.02	0.26 ± 0.02
A16	α	0.62 ± 0.05	0.62 ± 0.04	0.50 ± 0.04	0.55 ± 0.07	0.44 ± 0.03	0.50 ± 0.02	0.47 ± 0.04	0.44 ± 0.04
	β	0.46 ± 0.03	0.44 ± 0.03	0.51 ± 0.02	0.49 ± 0.04	0.51 ± 0.03	0.52 ± 0.04	0.51 ± 0.05	0.53 ± 0.04
F19	α	0.51 ± 0.03	0.53 ± 0.02	0.50 ± 0.03	0.56 ± 0.03	0.49 ± 0.02	0.53 ± 0.05	0.44 ± 0.02	0.48 ± 0.03
	β	0.33 ± 0.03	0.31 ± 0.03	0.33 ± 0.03	0.29 ± 0.03	0.26 ± 0.01	0.26 ± 0.03	0.25 ± 0.01	0.25 ± 0.02
	C1	0.53 ± 0.02	0.65 ± 0.05	0.55 ± 0.04	0.57 ± 0.04	0.54 ± 0.02	0.58 ± 0.01	0.63 ± 0.03	0.61 ± 0.05
	C2-C6	0.42 ± 0.01	0.39 ± 0.01	0.38 ± 0.01	0.38 ± 0.01	0.37 ± 0.01	0.37 ± 0.03	0.36 ± 0.01	0.37 ± 0.01
T22	β	0.49 ± 0.04	0.49 ± 0.02	0.43 ± 0.03	0.51 ± 0.04	0.43 ± 0.03	0.40 ± 0.01	0.47 ± 0.04	0.44 ± 0.02
	γ	0.32 ± 0.02	0.32 ± 0.02	0.32 ± 0.02	0.35 ± 0.02	0.40 ± 0.02	0.41 ± 0.04	0.43 ± 0.02	0.44 ± 0.02
G28	α	0.30 ± 0.02	0.29 ± 0.03	0.29 ± 0.03	0.31 ± 0.02	0.28 ± 0.03	0.27 ± 0.04	0.26 ± 0.01	0.25 ± 0.02
CO		0.96 ± 0.04	0.99 ± 0.06	1.05 ± 0.07	1.09 ± 0.05	1.10 ± 0.05	1.19 ± 0.03	1.15 ± 0.05	1.19 ± 0.04

Table S5: T_2^C relaxation time values (milliseconds) at sample temperatures from -5° C to 50° C, determined from single-exponential or stretched-exponential fits to the data in Fig. S14 and plotted in Fig. S19. For stretched-exponential fits, stretching exponents are given in parentheses. Identical values are listed for the S11 C_α and F19 C_α sites because signals from these sites were not resolved. A single set of values is given for backbone CO sites, which also did not have resolved signals. Uncertainties are standard errors from the fits.

^{13}C-T_2 (ms)		-5 °C	0 °C	5 °C	10 °C	20 °C	30 °C	40 °C	50 °C
L6	β	2.1 ± 0.6 (0.49 ± 0.09)	5.4 ± 1.1 (0.62 ± 0.12)	4.3 ± 1.3 (0.67 ± 0.21)	5.3 ± 1.2 (0.76 ± 0.19)	6.5 ± 0.8 (0.82 ± 0.14)	7.3 ± 0.8 (0.70 ± 0.10)	9.2 ± 0.7 (0.71 ± 0.06)	8.5 ± 0.4 (0.81 ± 0.05)
S11	α	4.6 ± 1.3 (0.60 ± 0.15)	8.1 ± 1.0 (0.59 ± 0.07)	12.1 ± 1.3 (0.62 ± 0.07)	11.5 ± 0.6 (0.68 ± 0.04)	14.8 ± 0.7 (0.68 ± 0.04)	18.3 ± 1.0 (0.88 ± 0.07)	23.5 ± 1.6 (0.81 ± 0.07)	28.0 ± 2.2 (0.91 ± 0.10)
	β	3.3 ± 0.7 (0.88 ± 0.26)	6.8 ± 0.9 (0.71 ± 0.10)	8.5 ± 0.9 (0.82 ± 0.11)	16.5 ± 1.7 (0.68 ± 0.08)	14.3 ± 1.7 (0.71 ± 0.11)	15.4 ± 1.4 (0.95 ± 0.14)	18.0 ± 1.3 (1.11 ± 0.14)	25.1 ± 2.0 (0.88 ± 0.12)
A16	α	2.6 ± 0.8 (0.57 ± 0.14)	7.5 ± 1.3 (0.76 ± 0.15)	7.4 ± 0.7 (0.83 ± 0.11)	7.2 ± 1.2 (0.74 ± 0.14)	8.9 ± 0.8 (0.98 ± 0.14)	13.2 ± 1.3 (1.07 ± 0.16)	24.5 ± 3.1 (0.84 ± 0.12)	33.2 ± 5.7 (0.78 ± 0.16)
	β	13.5 ± 0.7	9.1 ± 0.5	16.2 ± 1.4	15.1 ± 1.4	14.2 ± 0.8	39.4 ± 2.2	51.4 ± 3.1	53.6 ± 4.5
F19	α	4.6 ± 1.3 (0.60 ± 0.15)	8.1 ± 1 (0.59 ± 0.07)	12.1 ± 1.3 (0.62 ± 0.07)	11.5 ± 0.6 (0.68 ± 0.04)	14.8 ± 0.7 (0.68 ± 0.04)	18.3 ± 1.0 (0.88 ± 0.07)	23.5 ± 1.6 (0.81 ± 0.07)	28 ± 2.2 (0.91 ± 0.10)
	β	2.5 ± 0.6 (0.67 ± 0.15)	5.6 ± 0.9 (0.65 ± 0.11)	9.2 ± 1.2 (0.62 ± 0.09)	6.2 ± 0.5 (0.77 ± 0.08)	10.8 ± 1.1 (0.84 ± 0.12)	14.3 ± 1.7 (0.87 ± 0.13)	19.4 ± 1.1 (0.86 ± 0.06)	23.6 ± 1.6 (0.95 ± 0.10)
	C1	2.5 ± 0.2 (0.87 ± 0.09)	2.5 ± 0.1 (0.92 ± 0.06)	3.1 ± 0.3 (1.06 ± 0.18)	3.0 ± 0.3 (1.12 ± 0.17)	3.4 ± 0.3 (1.18 ± 0.20)	3.9 ± 0.4 (1.32 ± 0.31)	3.5 ± 0.2 (1.13 ± 0.14)	4.3 ± 0.4 (1.36 ± 0.30)
	C2-C6	2.2 ± 0.1 (1.02 ± 0.06)	2.6 ± 0.1 (1.23 ± 0.09)	3.1 ± 0.1 (1.21 ± 0.10)	3.3 ± 0.1 (1.35 ± 0.11)	4.3 ± 0.1 (1.54 ± 0.12)	4.7 ± 0.2 (1.87 ± 0.19)	4.7 ± 0.1 (1.81 ± 0.13)	5.4 ± 0.1 (1.95 ± 0.10)
T22	β	14.1 ± 1.8 (0.66 ± 0.09)	10.2 ± 1.3 (0.66 ± 0.09)	13.4 ± 1.5 (0.64 ± 0.07)	15.2 ± 1.3 (0.87 ± 0.10)	17.4 ± 1.3 (1.05 ± 0.12)	28.4 ± 3.6 (0.97 ± 0.16)	36.7 ± 8.7 (0.65 ± 0.13)	51.9 ± 6.2 (0.82 ± 0.12)
	γ	19.5 ± 2.0 (0.67 ± 0.08)	19.5 ± 1.3 (0.66 ± 0.06)	23.5 ± 2.8 (0.82 ± 0.11)	16.6 ± 0.6 (1.33 ± 0.08)	16.3 ± 0.9 (1.14 ± 0.10)	54.8 ± 4.9 (0.93 ± 0.12)	56.9 ± 2.3 (1.21 ± 0.08)	56.9 ± 2.4 (1.26 ± 0.10)
G28	α	3.0 ± 0.8 (0.61 ± 0.15)	5.6 ± 0.7 (0.58 ± 0.07)	9.3 ± 1.1 (0.60 ± 0.07)	7.0 ± 0.5 (0.80 ± 0.07)	10.4 ± 0.7 (0.77 ± 0.08)	18.2 ± 1.8 (0.75 ± 0.08)	22.6 ± 1.1 (0.78 ± 0.04)	42.5 ± 3.3 (0.70 ± 0.06)
CO		4.9 ± 0.5	5.4 ± 0.3	5.6 ± 0.4	8.4 ± 0.6	13.4 ± 1	19.9 ± 0.9	21.0 ± 0.9	33.0 ± 3.2
